# Structural variation and introgression from wild populations in East Asian cattle genomes confer adaptation to local environment

**DOI:** 10.1186/s13059-023-03052-2

**Published:** 2023-09-18

**Authors:** Xiaoting Xia, Fengwei Zhang, Shuang Li, Xiaoyu Luo, Lixin Peng, Zheng Dong, Hubert Pausch, Alexander S. Leonard, Danang Crysnanto, Shikang Wang, Bin Tong, Johannes A. Lenstra, Jianlin Han, Fuyong Li, Tieshan Xu, Lihong Gu, Liangliang Jin, Ruihua Dang, Yongzhen Huang, Xianyong Lan, Gang Ren, Yu Wang, Yuanpeng Gao, Zhijie Ma, Haijian Cheng, Yun Ma, Hong Chen, Weijun Pang, Chuzhao Lei, Ningbo Chen

**Affiliations:** 1 Key Laboratory of Animal Genetics, Breeding and Reproduction of Shaanxi Province, College of Animal Science and Technology, Northwest A&F University, Yangling, Xianyang China; 2 National Engineering Research Center for Non-Food Biorefinery, Guangxi Academy of Sciences, 98 Daling Road, Nanning, China; 3 Animal Genomics, ETH Zurich, Universitaetstrasse 2, 8006 Zurich, Switzerland; 4 The State Key Laboratory of Reproductive Regulation and Breeding of Grassland Livestock, School of Life Sciences, Inner Mongolia University, Hohhot, China; 5 Faculty of Veterinary Medicine, Utrecht University, Utrecht, The Netherlands; 6 Livestock Genetics Program, International Livestock Research Institute (ILRI), Nairobi, Kenya; 7 CAAS-ILRI Joint Laboratory On Livestock and Forage Genetic Resources, Institute of Animal Science, Chinese Academy of Agriculture Sciences (CAAS), Beijing, China; 8 Department of Infectious Diseases and Public Health, Jockey Club College of Veterinary Medicine and Life Sciences, City University of Hong Kong, Kowloon, Hong Kong SAR, China; 9 Tropical Crops Genetic Resources Institute, Chinese Academy of Tropical Agricultural Sciences, Haikou, China; 10 Institute of Animal Science & Veterinary Medicine, Hainan Academy of Agricultural Sciences, Haikou, China; 11 College of Veterinary Medicine, Northwest A&F University, Xianyang, Yangling China; 12 Qinghai Academy of Animal Science and Veterinary Medicine, Qinghai University, Xining, China; 13 Institute of Animal Science and Veterinary Medicine, Shandong Academy of Agricultural Sciences, Shandong Key Lab of Animal Disease Control and Breeding, Jinan, China; 14 Key Laboratory of Ruminant Molecular and Cellular Breeding of Ningxia Hui Autonomous Region, School of Agriculture, Ningxia University, Yinchuan, China

**Keywords:** Structural variation, Genome assembly, Long-read sequencing, East Asian cattle

## Abstract

**Background:**

Structural variations (SVs) in individual genomes are major determinants of complex traits, including adaptability to environmental variables. The Mongolian and Hainan cattle breeds in East Asia are of taurine and indicine origins that have evolved to adapt to cold and hot environments, respectively. However, few studies have investigated SVs in East Asian cattle genomes and their roles in environmental adaptation, and little is known about adaptively introgressed SVs in East Asian cattle.

**Results:**

In this study, we examine the roles of SVs in the climate adaptation of these two cattle lineages by generating highly contiguous chromosome-scale genome assemblies. Comparison of the two assemblies along with 18 Mongolian and Hainan cattle genomes obtained by long-read sequencing data provides a catalog of 123,898 nonredundant SVs. Several SVs detected from long reads are in exons of genes associated with epidermal differentiation, skin barrier, and bovine tuberculosis resistance. Functional investigations show that a 108-bp exonic insertion in *SPN* may affect the uptake of *Mycobacterium tuberculosis* by macrophages, which might contribute to the low susceptibility of Hainan cattle to bovine tuberculosis. Genotyping of 373 whole genomes from 39 breeds identifies 2610 SVs that are differentiated along a “north–south” gradient in China and overlap with 862 related genes that are enriched in pathways related to environmental adaptation. We identify 1457 Chinese indicine-stratified SVs that possibly originate from banteng and are frequent in Chinese indicine cattle.

**Conclusions:**

Our findings highlight the unique contribution of SVs in East Asian cattle to environmental adaptation and disease resistance.

**Supplementary Information:**

The online version contains supplementary material available at 10.1186/s13059-023-03052-2.

## Background

Cattle are one of the most important livestock species in the world, as they provide important resources (e.g., meat, milk, hides, and draught power) to humans. Modern domestic cattle belong to two cross-fertile subspecies, including the humped indicine cattle (*Bos taurus indicus*) and humpless taurine cattle (*Bos taurus taurus*), which originated from a distinct aurochs *Bos primigenius* subspecies with an ancestral divergence time from ~ 200 thousand years ago to less than 1 million years ago in South and Southwest Asia [1–4]. After their domestication, they spread across the world following human migration and trading, leading to their genetic adaptations to different environmental conditions, including extremely cold and hot climates [5, 6].

Long-term environmental adaptation as well as recent interspecies introgression and artificial selection have contributed to pronounced genomic diversity among breeds of East Asian cattle. The Chinese indigenous cattle breeds became adapted to various agroecological conditions within the vast territory of China. The northern Chinese breeds, represented by Mongolian cattle, are mostly of taurine origin and well adapted to a cold climate [7]. The southern Chinese breeds, including the Hainan cattle, are predominantly of indicine origin but are genetically distinct from other indicine populations such as South Asian and African indicine cattle [5], following potential introgression from banteng (*Bos javanicus*) or other unsampled bovine species in East Asia [5, 8], which may have facilitated a rapid adaptation of Chinese indicine cattle to hot and humid environments with high pathogen burden and strong ultraviolet radiation (UV) exposure [9].

The genetic variation of East Asian cattle breeds has been extensively characterized using single nucleotide polymorphisms (SNP) [5]. However, structural variations (SV), including insertions (INS), deletions (DEL), inversions (INV), and duplications (DUP), are considered to have a greater impact on gene expression than SNPs [10, 11]. Some SVs have been identified to underpin the so-called domestication traits of animals [12]. For instance, recent characterization of the Angus and Brahman cattle genomes identified SVs related to indicine immunity (disease challenge and external parasites) and fatty acid desaturase [13]. Similar studies on East Asian cattle are hampered by inherent limitations of the short-read sequencing (SRS) data and their strong genetic divergence from current taurine reference genome to propagate a reference allele bias [5, 14]. Reference-quality genomes have been assembled for a Hereford cattle of European taurine origin and a Brahman cattle of taurine × indicine hybrid origin [13, 15, 16], but not yet for any East Asian cattle, which precludes unbiased SVs discovery.

**Fig. 1:**
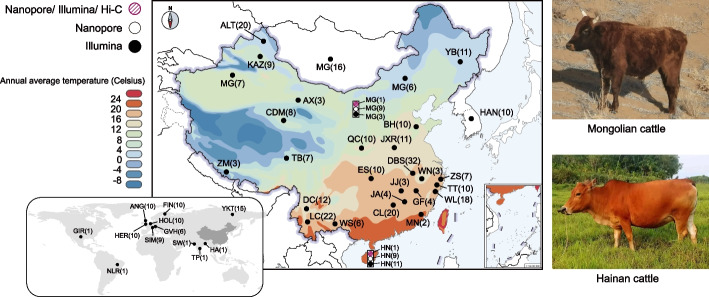
Geographical distribution of the 39 cattle breeds/populations used in this study. Brackets indicate the number of individuals of the breed/population. The rectangle indicates that this breed/population has data from three sequencing platforms. The photos show Mongolian and Hainan cattle with their representative habitats. AGS, Angus; ALT, Altay; AX, Anxi; BH, Bohai Black; CDM, Chaidamu; CL, Chaling; DBS, Dabieshan; DC, Dengchuan; ES, Enshi; FIN, Finncattle; GF, Guangfeng; GIR, Gir; GVH, Gelbvieh; HA, Hariana; HAN, Hanwoo; HER, Hereford; HN, Hainan; HOL, Holstein; JA, Ji’an; JJ, Jinjiang; JXR, Jiaxian Red; KAZ, Kazakh; LC, Lincang; MG, Mongolian; MN, Minnan; NLR, Nelore; QC, Qinchuan; SIM, Simmental; SW, Sahiwal; TB, Tibetan; TP, Tharparkar; TT, Tiantai; WL, Wenling; WN, Wannan; WS, Wenshan; YB, Yanbian; YKT, Yakutian; ZM, Zhangmu; ZS, Zhoushan

In this study, we assembled two high-quality chromosome-level genomes of East Asian taurine (Mongolian_v1) and indicine (Hainan_v1) cattle using a mixture of long-read sequencing (LRS) and SRS as well as chromatin conformation capture (Hi-C) data. Furthermore, based on novel LRS data of nine Hainan and nine Mongolian cattle as well as SRS data of 373 cattle from 39 geographically dispersed breeds (Fig. 1), we revealed the geographic allele distributions of 123,898 nonredundant SVs in the northern and southern Chinese cattle breeds and identified SVs alleles originated from the banteng genomes in the southern Chinese cattle breeds. We found a set of unique SVs related to specific genes responsible for skin differentiation, heat and pathogen tolerance, and immune response that contribute to regional adaptations to the hot and humid southern Chinese climate.

## Results

### Genome sequencing, assembly, quality control, and annotation

We assembled the genomes of a male Hainan (Hainan_v1) and a male Mongolian (Mongolian_v1) cattle with a mixture of LRS (Hainan 285 Gb and Mongolian 335 Gb) and SRS (Hainan 63 Gb and Mongolian 92 Gb) data produced by the Oxford Nanopore and Illumina sequencing platforms, respectively (Additional file 1: Fig. S1 and Additional file 2: Table S1). The initial hybrid assemblies had contig N50 values of 44.35 Mb for Hainan_v1 and 47.79 Mb for Mongolian_v1, and further scaffolding with ~ 100 × coverage Hi-C data generated scaffolds with N50 values of 104.22 Mb and 104.05 Mb, respectively (Additional file 1: Figs. S2 and S3). The overall sizes of the Hainan_v1 and Mongolian_v1 were 2.652 Gb and 2.638 Gb, respectively (Table 1).

**Table 1:** Quality statistics of the assembled Hainan and Mongolian cattle genomes

Statistics	Hainan_v1	Mongolian_v1	ARS_UCD1.2
Genome size (bp)	2,651,892,370	2,638,491,090	2,715,853,792
Contig N50 (bp)	44,352,259	47,790,958	25,896,116
Scaffold N50 (bp)	104,224,000	104,053,164	103,308,737
Number of scaffolds	301	400	2211
Max scaffold (bp)	157,081,543	146,429,245	158,534,110
Min scaffold (bp)	749	1000	1034
Scaffold mean (bp)	8,810,273	6,596,227	1,228,337
Repetitive sequences (%)	46.56%	46.60%	45.73%
Consensus quality score (QV)^a^	36.66	40.58	37.11

^a^Numbers in QV indicate the values in chromosome scaffolds only, evaluated by using Merqury [17]

Four methods were used to assess the quality of these two novel assemblies. First, the Benchmarking Universal Single-Copy Ortholog (BUSCO) [18] analysis based on the mammalia_odb9 database showed that 93.8% and 93.2% of single-copy genes were identified in Hainan_v1 and Mongolian_v1, respectively (Additional file 2: Table S2). This was similar to the latest Angus (92.9%) and Brahman (93.5%) assemblies [13]. Second, the alignment of novel scaffolds against the current taurine reference genome (ARS_UCD1.2, GCF_002263795.1) demonstrated high collinearities of Hainan_v1 and Mongolian_v1 (Additional file 1: Figs. S4-S6). Third, we mapped the SRS data back to the two assemblies and found that 97.28% and 94.78% of the Hainan and Mongolian SRS data were mapped to Hainan_v1 and Mongolian_v1 with average depths of 18.09 × and 25.59 × , respectively (Additional file 1: Fig. S7 and Additional file 2: Table S3). Fourth, the analysis by Merqury [17] revealed consensus quality scores (QVs) of 36.66 for Hainan_v1 and 40.58 for Mongolian_v1 (Table 1).

There were 46.6% of interspersed repeats in both Hainan_v1 and Mongolian_v1 (Additional file 2: Tables S4-S5), with long interspersed (LINE, 27.9%) and short interspersed nuclear elements (SINE, 11.7%) as the most important categories. A total of 25,763 and 25,924 protein-coding genes were predicted in Hainan_v1 and Mongolian_v1, covering 90.4% and 92.1% of their complete BUSCOs, respectively (Additional file 2: Table S2).

### Advantage of population-specific assemblies in sequence alignment and variant calling

Previous studies have shown that a population-specific assembly improved the mapping rate of SRS data [19]. For this evaluation, we aligned the SRS data from the HN024 Hainan and NMG016 Mongolian samples to three assemblies (Hainan_v1, Mongolian_v1, and ARS_UCD1.2) using the Burrows–Wheeler Aligner (BWA) [20]. As expected on the basis of previous observations in human genomes [19], the two Asian assemblies exhibited a 5–7% better mapping rate than the European taurine assembly (ARS_UCD1.2) across a series of mapping quality (MQ) thresholds (Additional file 1: Fig. S8), while the lowest number of SNPs was identified in HN024 and NMG016 if they were aligned to Hainan_v1 and Mongolian_v1, respectively.

The effect of the reference assembly was also observed if the SRS data from 373 samples representing 39 breeds were aligned to the three assemblies of Hainan_v1, Mongolian_v1, and ARS_UCD1.2 (Additional file 2: Table S6 and S7). However, the alignments to ARS_UCD1.2 showed fewer SNPs than the alignments to Mongolian_v1, due likely to the higher quality of ARS_UCD1.2 with a stronger impact on variant detection than the allelic bias [19] (Table 1 and Additional file 2: Table S2). In addition, the highest number of SNPs was identified across Chinese indicine cattle, a further indication of their higher genetic variation.

### SV detection in Hainan and Mongolian cattle based on whole-genome alignment and LRS data

**Fig. 2:**
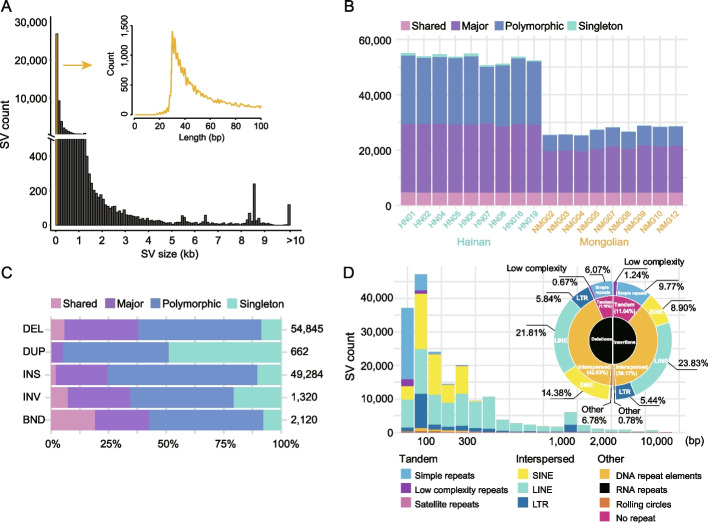
Discovery of structural variations in Hainan and Mongolian cattle. **A** Sizes of SVs identified by pairwise alignment of the Hainan_v1 and Mongolian_v1 assemblies. **B** Number of SVs per sample that are classified into shared (identified in all samples), major (in ≥ 9 samples), minor (in > 1 sample), and singleton (in only one sample) SVs. **C** Proportions of SVs in the four categories defined in **B**. **D** Distribution of insertions and deletions of different interspersed repeat elements

We first compared Mongolian_v1 (as reference) and Hainan_v1 by whole-genome alignment as performed previously [21], which yielded 55,131 SVs, including 27,615 INSs, 26,834 DELs, 502 DUPs, and 180 INVs. In the 54,449 INDELs (INSs and DELs), the lengths of SVs ranged from 10 bp to 96 kb. As expected, the majority of the INDELs were short (38.6% = 30–40 bp, 48.99% < 100 bp, and only 0.21% > 10 kb) (Additional file 2: Table S8 and Fig. 2A).

The comparison of Hainan_v1 and Mongolian_v1 only captured partial SVs in the Hainan and Mongolian cattle breeds. Additional SVs were therefore identified by generating LRS data from nine Hainan and nine Mongolian cattle, which were mapped on Mongolian_v1. An average sequence coverage of 10.80 × (5.72–16.59) (Additional file 2: Table S9) provided a sufficient depth for the detection of 112,451 SVs that were supported by at least two methods for SVs discovery (CuteSV [22], SVIM [23], and Sniffles [24]). After filtering out 4220 unreliable genotypes, 108,231 SVs were retained, including 54,845 DELs, 49,284 INSs, 662 DUPs, 1320 INVs, and 2120 complex structural variants (BND) (Additional file 2: Table S10). We compared the numbers of SVs obtained based on the whole-genome alignment and LRS data (11.02 ×) from the same samples of HN024 for Hainan_v1 and NMG016 for Mongolian_v1 and identified 38,254 (19,765 INSs and 18,489 DELs) and 23,554 (7974 INSs and 15,580 DELs) INDELs > 50 bp, respectively. A total of 17,756 SVs were overlapped between the two methods, including 6293 INSs and 11,463 DELs. The higher INDELs from the whole-genome alignment were attributed to the higher accuracy of assembly-based SVs [25], which is consistent with previous results [26]. More DELs were observed in the LRS data due possibly to base-calling errors of homopolymer regions, a major drawback of Nanopore sequencing [27].

We classified the LRS-based SVs into four categories: shared (identified in all samples, *n* = 18), major (in ≥ 50% of samples, *n* = 9 to 17), minor (in > 1 sample, *n* = 2 to 8), and singleton (in only one sample, *n* = 1) SVs. Approximately 31.6% (*n* = 34,164) of the LRS-based SVs were shared or major, which indicates that the Mongolian_v1 reference genome carries a minor allele or an error at these locations as observed previously for human assemblies [28]. Among the shared SVs, DELs were more abundant than INSs (Fig. 2B, C), which was also consistent with the results obtained in humans [29].

Differences in allele frequencies between populations may reveal divergent alleles or selective variants [30]. We calculated the fixation index (*F*_ST_) to assess the differences in LRS-based SVs and search for divergent SVs between the Hainan and Mongolian cattle breeds [31]. Based on the significant differentiation between cattle breeds from northern (*B. t. taurus*) and southern (*B. t. indicus*) China [14], we defined *F*_ST_ > 0.25 as population-differentiated SVs [31]. A total of 41,435 Hainan-Mongolian differentiated SVs were identified, including 20,000 INSs, 20,818 DELs, 317 INVs, and 300 DUPs (Additional file 2: Table S11). ANNOVAR [32] annotation of these SVs in Mongolian_v1 showed that 14,694 (35.5%) of them were within or near genes, including 269, 267, 2, 13,965, and 191 SVs in 1-kb upstream regions, exons, splicing signals, introns, and UTRs, respectively (Additional file 2: Table S12).

Three of these SVs were present in the exons of three genes that were associated with epidermal differentiation (*CRNN* and *SBSN*) and skin barrier (*SPINK5*) (Table 2 and Additional file 1: Fig. S9). A 126-bp DEL in the third exon of *CRNN* (Additional file 1: Fig. S9A), a member of the epidermal differentiation complex [33], showed a higher frequency in Hainan cattle (0.85) than in Mongolian cattle (0.05) (Additional file 1: Fig. S9D). A 54-bp DEL in the first exon of *SBSN* has been implicated in regulating skin differentiation and the skin barrier [34] (Additional file 1: Fig. S9B) and was found only in Hainan cattle with a frequency of 0.65 (Additional file 1: Fig. S9D). *SPINK5* is involved in the regulation of proteolysis in epithelial formation and keratinocyte terminal differentiation, whereas mutations in human *SPINK5* cause a distinctive defect in skin barrier function in Netherton syndrome [35]. A 988-bp DEL in exons 10 to 12 of *SPINK5* was observed only in Hainan cattle with a frequency of 0.60 (Additional file 1: Figs. S9C and D).

**Table 2:** Candidate genes linked with the “Hainan-Mongolian” differentiated SVs

No. of location (Mongolian_v1)	Type	Length (bp)	Region	*F*_ST_	Gene	Trait
BTA03:18,452,000–18,452,126	Deletion	126	Exon 3	0.77	*CRNN*	Epidermal differentiation
BTA07:51,555,138–51,556,126	Deletion	988	Exon 1	0.67	*SPINK5*	Skin barrier
BTA18:19,542,094–19,542,148	Deletion	54	Exons 10–12	0.62	*SBSN*	Epidermal differentiation;
						skin barrier
BTA25:26,642,984–26,642,984	Insertion	108	Exon	0.78	*SPN*	Disease resistance

*Note*: The locations and lengths of the variations were based on the alignment of the Hainan_v1 and Mongolian_v1 genomes

*F*_ST_, fixation index between the Hainan and Mongolian cattle breeds

### Function of an insertion in the exon of *SPN* gene for binding of *Mycobacterium tuberculosis*

**Fig. 3:**
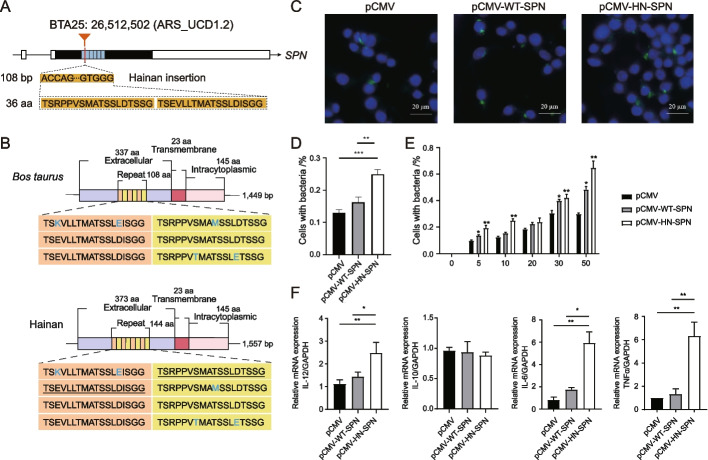
Effects of the 108-bp insertion in the exon of the *SPN* gene in Hainan cattle on *Mycobacterium tuberculosis*. **A** Schematic representation showing the chromosomal position of the 108-bp insertion and the partial sequences of the 108-bp insertion obtained by genome alignment. **B** Schematic representation of the CD43 protein structures of taurine (WT-SPN) and Hainan (HN-SPN) cattle. **C**, **D** The RAW264.7 cells were transfected with pCMV-WT-SPN or pCMV-HN-SPN for 48 h and then treated with the H37Ra strain (MOI = 0, 5, 10, 20, 30, and 50) for 4 h. The H37Ra strain was detected by immunofluorescence. **E** The RAW264.7 cells were transfected with pCMV-WT-SPN or pCMV-HN-SPN for 24 h and then treated with the H37Ra strain (MOI = 10) for 24 h. **F** The mRNA levels of IL-12, IL-10, IL-6, and TNFα were determined using qRT-PCR

Indicine cattle have evolved a stronger genetic resistance to ticks, parasite transmission, and other tropical diseases than taurine cattle [36]. We identified a specific 108-bp INS in the single exon of *SPN* (sialophorin, CD43) that encodes with an additional repeat of 36 amino acids in its extracellular domain (Fig. 3A, B). This INS structure was validated by both IGV (Additional file 1: Fig. S10) and PCR (Additional file 1: Fig. S11). Other bovine species had only four to six of such repeats (Additional file 1: Fig. S12). CD43 was found to be associated with the antigen-specific activation of T cells [37] and to inhibit the growth of *Mycobacterium tuberculosis* in humans [38]. CD43-deficient mice had a reduced ability to control *M. tuberculosis* growth during the acute and chronic phases of infection [38].

The CD43 extracellular domain has a rod-like structure that is rich in serine or threonine residues modified by heavily sialylated O-linked glycans and is extended from the macrophage cell surface, leading to mycobacterial-specific recognition through binding to the *M. tuberculosis* capsule protein Cpn60.2 [38, 39]. To investigate whether the increased number of O-linked glycosylation sites in CD43 affected the uptake of *M. tuberculosis* by macrophages, we transfected WT-SPN (wild-type, pCMV-WT-SPN) or HN-SPN (Hainan-type, pCMV-HN-SPN) into the RAW264.7 (ECACC 91062702) macrophage cell line 24 h before the infection by the H37Ra strain of *M. tuberculosis* at a range of multiplicity of infection (MOI) for 4 h (Fig. 3C). We found that the overexpression of CD43 resulted in significantly enhanced uptakes of *M. tuberculosis* by the pCMV-HN-SPN relative to the pCMV-WT-SPN (Fig. 3D) across a wide range of MOI at 5:1, 10:1, 20:1, 30:1, and 50:1 (Fig. 3E). Similar to the observation that CD43 absence reduced the production of TNF-α, IL-12, and IL-6 in the macrophages infected by *M. tuberculosis* [40], we detected significantly higher TNF-α and IL-6 in the RAW264.7 cells transfected by pCMV-HN-SPN compared with pCMV-HN-SPN, after the infection by H37Ra strain at an MOI of 10:1 (Fig. 3F). Because TNF-a is crucial in restricting the growth of *M. tuberculosis*, we speculated that the 108-bp INS in the *SPN* gene was related to the low susceptibility of indicine cattle to bovine tuberculosis.

### Genotyping of SRS-based SVs

We merged the whole-genome alignment-based and LRS-based SVs for INSs and DELs and compiled a set of 123,898 nonredundant SVs, including 64,622 DELs and 59,276 INSs (Additional file 2: Table S10). These SVs were enriched in DELs and INSs around 300 bp or 1000 bp (Fig. 2D and Additional file 1: Fig. S13) corresponding to SINEs (BOV-A2) and LINEs, respectively. We also found that 20.4% of the SVs, mostly in INSs, were overlapped with the L1_BT LINE around 300 bp.

We used the Paragraph program [41] to genotype the combined set of SVs in the SRS data of 373 cattle from 39 geographically dispersed breeds, including 288 individuals from 26 Chinese cattle breeds represented by eight northern taurine breeds/populations (Mongolian, Chaidamu, Yanbian, Altay, Anxi, Zhangmu, Tibetan, and Kazakh), 13 southern indicine breeds/populations (Hainan, Guangfeng, Ji’an, Jinjiang, Tiantai, Wenling, Minnan, Wannan, Dabieshan, Lincang, Wenshan, Chaling, and Zhoushan), and five hybrid cattle breeds from central China (Bohai Black, Jiaxian Red, Qinchuan, Enshi, and Dengchuan) (Fig. 1 and Additional file 2: Table S13). We found that 93.7% (116,125/123,898) of the SVs were genotyped in 80% of the 373 cattle, of which 94.8% (110,129/116,125) were genotyped in only one cattle. This 10% or higher missing genotyping rate in the SRS data for the SVs discovered by whole-genome alignment and LRS data was observed in a previous study [41] (Additional file 1: Fig. S14).

**Fig. 4:**
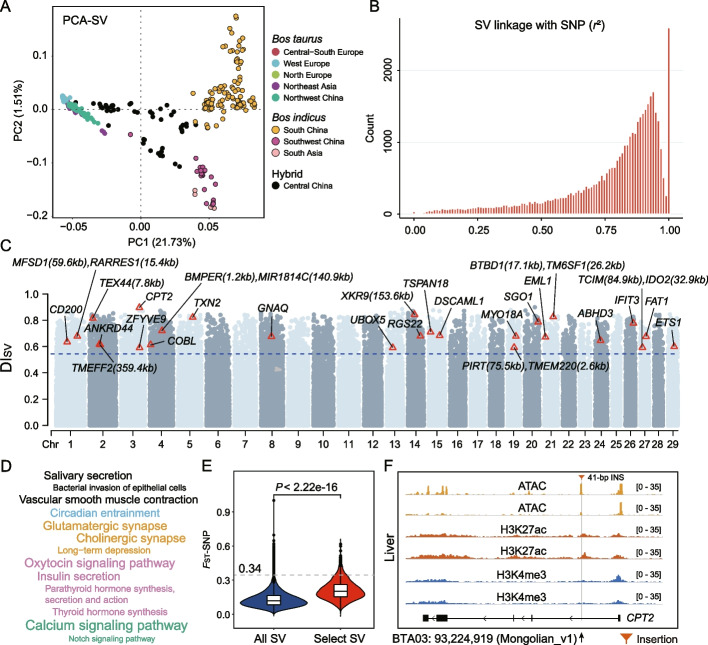
Population genetic differentiation and functional analysis of SRS-based SVs. **A** Principal component analysis of the 39 cattle breeds/populations based on the SVs. **B** Linkage analysis between SVs and surrounding SNPs (300 SNPs flanking the SVs). **C** Manhattan plot of the DI_SV_ statistics between northern and southern Chinese cattle based on the SVs. The dashed blue line indicates the top 5% proportion of SVs with DI_SV_. The red triangle represents the SVs belonging to the top 5% signals from both DI_SV_ and *F*_ST_-SV and falling in the ATAC peaks. The distances of SVs from the genes are listed in brackets. **D** Significantly enriched KEGG pathways (corrected *P* < 0.01) for the genes that are structurally linked to the stratified SVs. **E** Distribution of the mean *F*_ST_ values of the SNPs (*F*_ST_-SNPs) surrounding the selected SVs compared with all SVs in a 5-kb window. The dashed line indicates the top 1% cutoff of *F*_ST_-SNP distributions around all SVs. **F** An example of a 41-bp insertion (INS) (BTA03: 93,224,919, Mongolian_v1) in the enhancer of the *CPT2* gene with a high allele frequency difference (DI_SV_ = 0.897) and *F*_ST_-SV (0.889) between northern and southern Chinese cattle. The numbers in brackets in the picks of ATAC-seq and ChIP-seq (H3K4me3 and H3k27ac) data indicate signal intensities

After filtering out the SVs with minor allele frequency less than 0.05, 61,513 qualified SVs were used to characterize the population stratification among the 373 cattle. Principal component analysis (PCA) clearly distinguished cattle from different geographical regions (Fig. 4A) and mirrored their phylogenetic relationship based on genome-wide SNPs (Additional file 1: Fig. S15). PC1 divided the taurine from indicine ancestries; the indicine cattle from South Asia, South China, and Southwest China were differentiated from each other at PC2, while the hybrid cattle from central China were located between the taurine and indicine ancestries. The neighbor-joining (NJ) phylogenetic tree and ADMIXTURE analysis validated the pattern of PCA (Additional file 1: Figs. S16 and S17).

We investigated the intensity of linkage between individual SVs and surrounding SNPs (300 SNPs flanking the SVs) and realized that only 62% of the SVs had high linkage disequilibrium (LD) with nearby SNPs (*r*^2^ > 0.8), suggesting the presence of hidden genetic variants that were only captured by SNPs (Fig. 4B).

### Population genetic differentiation and functional analysis of SRS-based SVs

A previous study based on genome-wide SNPs has found selection signals in cattle from northern and southern China in response to local environmental challenges [14]. To investigate candidate SVs that have shaped the unique adaptation of Chinese cattle, we used a combination of DI_SV_ and *F*_ST_ (*F*_ST_-SV) methods to identify population-stratified SVs based on the SRS data at 5% signals of both DI_SV_ (DI_SV_ = 0.54) and *F*_ST_-SVs (*F*_ST_ = 0.52) between the northern and southern Chinese cattle (Fig. 4C) and identified 2610 SVs to be involved in the “north–south” stratification. Among them, 1070 SVs were found to be linked with 862 genes based on their presence in exons (10), UTRs (18), upstream (< 1 kb, 14), intergenic regions (< 3 kb, 27), and introns (1001). Functional enrichment analyses of the 862 genes using Kyoto Encyclopedia of Genes and Genomes (KEGG) and Gene Ontology (GO) showed their significant enrichments in pathways (corrected *P* < 0.05) related to environmental adaptation (circadian entrainment), immunity (bacterial invasion of epithelial cells), and endocrine system (insulin secretion, oxytocin signaling pathway, parathyroid hormone synthesis, secretion and action, and thyroid hormone synthesis) (Fig. 4D and Additional file 2: Table S14).

In addition, we performed an *F*_ST_ analysis using the genome-wide SNPs from the same populations to see how many genes identified from top *F*_ST_-SNP values (e.g., top 5%) were overlapped with *F*_ST_-SV genes. Up to 61%, 53%, 44%, 32%, and 25% of the genes were found in the non-overlapping sliding windows of 5 kb, 10 kb, 20 kb, 50 kb, and 100 kb, respectively. We evaluated whether the SNPs surrounding the 2610 stratified SVs were also differentiated based on the *F*_ST_-SNPs in 5-kb non-overlapping sliding windows and observed that averaged *F*_ST_-SNPs values surrounding these stratified SVs were significantly higher than those surrounding all SVs (120,006 autosomal SVs, Wilcoxon rank-sum test *P* < 2.22e − 16). However, the *F*_ST_-SNPs surrounding the most stratified SVs (2608, 99.92%) were below the 90th quantile (0.57) of the *F*_ST_-SNPs (Fig. 4E), indicating that these SVs may probably be missed by traditional SNPs-based analyses.

### Integrative of ATAC-seq and ChIP-seq data

A large part of the SVs were located in the noncoding sequences, including promoters and UTRs, so they may be associated with the expression of nearby genes [11]. To explore the potential regulatory roles of the 2610 stratified SVs, we integrated independent epigenetic data (i.e., ATAC-seq and ChIP-seq for H3K4me3 and H3k27ac histone marks) from seven tissues (lung, liver, spleen, muscle, cerebral cortex, hypothalamus, and adipose) of cattle. Using the ATAC-seq data, we identified 117,789 peaks for lung, 77,335 for muscle, 43,021 for liver, 22,839 peaks for spleen, 37,039 for cerebral cortex, 9417 for hypothalamus, and 15,527 for adipose, of which 23, 8, 6, 1, 4, 1, and 0 were overlapped with some of these SVs, respectively (Additional file 2: Table S15). *CD200* on *Bos taurus* autosome (BTA) 01 and *CPT2* on BTA03 were chosen as examples to illustrate the signal distributions of SVs in the ATAC-seq and ChIP-seq picks (Fig. 4F and Additional file 1: Fig. S18). We identified a 64-bp deletion and a 41-bp insertion in the intronic regions of *CD200* and *CPT2*, respectively, both were located in the enhancer regions identified by the ChIP-seq data. *CD200* (formerly known as *OX2*) is implicated in the delivery of immunosuppressive signals to the immune system [42, 43]. *CPT2* encodes carnitine palmitoyltransferase 2, which is critical for fatty acid metabolism [44]. Immunity and fatty acid metabolism were probably involved in environmental adaptation. Further studies are needed to elucidate the underlying molecular mechanisms of these SVs in regulatory regions in the genomes of northern and southern Chinese cattle.

### Population-stratified SVs derived from banteng introgression into Chinese indicine cattle

Stratified SVs between populations may either emerge within the populations or represent the combined results of gene flow from related species living in the same geographic area and under selection pressure [30]. Although our previous study showed that historic introgression events have helped southern Chinese indicine cattle to achieve a rapid adaptation by acquiring ~ 2.93% of their genetics from banteng [5], we genotyped our SVs in three high-coverage banteng genomes (Additional file 2: Table S13) and then searched for the SVs that were shared by the southern Chinese indicine cattle and banteng but absent in 55 European taurine and five South Asian indicine cattle (see the “Methods” section). To reduce the effects of incomplete lineage sorting and complex evolutionary relationships across wild bovine species in Asia [8], we also assumed a complete absence of such shared SVs in gaur genomes (*Bos gaurus*). Up to 1466 SVs specific to Chinese indicine cattle were identified as probably introgressed from banteng, with their lengths ranging from 28 bp to 24 kb in linkage with 549 genes. Based on the RFMix analysis results, we found that nearly all these SVs (1457/1466) fell into the introgressed genomic regions screened by genome-wide SNPs, an indication of their credibility.

To identify the potential adaptively introgressed SVs in Chinese indicine cattle genomes, we searched for common and highly “north–south” stratified SVs (*F*_ST_-SV > 0.25) in the 1457 overlapped genomic regions, yielding 24 SVs linked with eight genes (Additional file 2: Table S16), which were located in intergenic (15), intronic (8), and ncRNA_intronic (1) regions and may play some roles in the regulation of linked genes. One of the banteng-shared SV alleles with a frequency of 53% in southern Chinese cattle was a 316-bp DEL (BTA08:11,360,524–11,360,840, Mongolian_v1) within the intron of *DDX58*, an RNA helicase gene (Additional file 1: Fig. S19). *DDX58* encodes retinoic acid-inducible gene I (RIG-I), which is important to innate immune defense against viruses [45] and involved in the specific recognition of hantavirus, rotavirus, and filovirus [46].

## Discussion

The most widely used cattle reference genome assembly is from a European Hereford cow, while the recent bovine pangenomes used assemblies from European taurine, the taurindicine (~ 90% indicine) Brahman, the African cattle, and the yak [16, 47, 48]. Considering the unique genomic diversity of East Asian cattle, which are different from European taurine and South Asian indicine cattle, as well as the influence of the genetic introgression of wild bovine species, the availability of reference genomes for East Asian cattle will be essential to accurately reveal the genetic diversity of East Asian cattle genetic resources, particularly the SVs in the genomes of East Asian cattle. Here, we present two reference-quality genome assemblies of cattle adapted to two contrasting climates in China, the Mongolian taurine and the Hainan indicine cattle breeds (Additional file 1: Fig. S6 and Table 1). These assemblies may serve as references for some Asian cattle breeds and contribute to a more comprehensive bovine pangenome.

Reference-guided alignment of SRS data does not allow for comprehensive detection of SVs [49, 50]. In this study, we leveraged assemblies and long sequencing reads to provide a comprehensive overview of the SVs that are prevalent in the Mongolian and Hainan cattle genomes. From the final nonredundant set of the SVs, 88.9% of them were called in SRS data from a wider panel of cattle breeds/populations. The missing rate was higher in southern cattle than in northern cattle (Additional file 1: Fig. S14), which probably reflected that SVs genotyping with SRS data [29] can be confounded by high heterozygosity of East Asian indicine cattle.

For functional characterization of the LRS-based SVs, we focused on Hainan-Mongolian differentiated SVs in potential functional regions and positioned within exons. A previous comparison between the Brahman and Angus cattle found additional copies of the *FADS2P1* gene in Brahman, which may be involved in altering skin water permeability and heat loss [13]. In this study, three SVs associated with epidermal differentiation (*CRNN* and *SBSN*) and skin barrier (*SPINK5*) were identified in Hainan and Mongolian cattle, possibly reflecting the differences in environmental adaptation between indicine and taurine cattle based on their unique skin morphology, hair follicle density, and sweat gland area and density [51, 52]. Bovine tuberculosis is a zoonosis mainly caused by the inhalation of infectious particles from *Mycobacterium bovis* and particularly problematic if local cattle are kept at high density in developing countries [53]. The prevalence and severity of the pathology of bovine tuberculosis are higher in taurine than in indicine cattle or taurindicine crossbreds [54, 55]. Notably, we identified a specific 108-bp INS that created additional copies of a 36-amino acid repeat in the extracellular domain of CD43. The CD43 extracellular domain has a rod-like structure that is predicted to extend from the cell surface and contains serine or threonine residues, most of which are modified with heavily sialylated O-linked glycans. We hypothesize that the 108-bp INS will increase the number of sites for O-linked glycosylation, further increasing the ability to bind CD43 and inhibit the growth of *M. tuberculosis*, which plausibly causes a lower susceptibility to bovine *tuberculosis* in indicine cattle [38]. These population-differentiated SVs are related to skin differentiation, heat resistance, and pathogen resistance. Considering the adaptation of East Asian indicine cattle to humid and hot environments, we infer that the SVs of these genes contribute to the local environmental and disease adaptation of East Asian indicine cattle. However, more evidence is needed to further characterize the impact of these SVs. Some of these SVs such as duplications in the *SPN* gene are inaccessible from short read alignments, illustrating the benefits of identifying SVs at the population level with LRS data. As sequencing technologies develop, third-generation sequencing data will continue to become more cost-effective and accessible, making the population-scale capture of SVs more faithful and feasible.

We used a large amount of SRS data to genotype the SVs identified in Hainan and Mongolian cattle and identify the SVs that differ between northern and southern Chinese cattle. Although the vast majority of stratified SVs are noncoding variants, they may influence the expression of linked genes. To detect candidate SVs in regulatory regions, we collected data on open chromatin regions of Hereford cattle for the ATAC-seq and ChIP-seq analyses. Although these data are only available for the taurine cattle, it allows a tentative dentification of conserved regulatory regions [56]. We anticipate that additional collection of epigenetic data will help to accurately identify the regulatory SVs conferring the environmental adaptation of cattle.

Our previous study revealed that southern Chinese indicine cattle achieved a rapid adaptation by acquiring banteng ancestry [5]. In humans, it has been reported that high-quality de novo assembly can be used to search for introgressed sequences from other species but is absent in the reference genome [49]. In this study, using the Illumina data from banteng with high sequencing coverage, we generated a high-quality dataset of 1457 SVs that were introgressed from banteng into Chinese indicine cattle. Due to the lack of a high-coverage *Bos sauveli* (kouprey, possibly extinct) genome [8], it is impossible to detect kouprey introgression into Chinese indicine cattle. Our results showed that 99.4% of the introgressed SVs were within the regions of introgressed SNPs, confirming our identification of the introgressed SVs. Taking 316-bp DEL in the *DDX58* gene as an example, we constructed a phylogenetic tree in 46 individuals to be homozygous for the introgressed SVs, to infer the origin of haplotypes based on the SNPs surrounding the SVs (Additional file 1: Fig. S19). The results showed that the 316-bp DEL was indeed located within the region of banteng-introgressed SNPs. However, the origin of southern Chinese cattle is rather complex, the impact of other bovine species on Chinese indicine cattle cannot be completely excluded, including the extinct (kouprey) or other unsampled wild bovine species or lineages [5, 8, 57].

## Conclusions

In conclusion, our results highlight the important roles of SVs in the adaptation of cattle subspecies to local environmental challenges in China. We provide insights into the functional roles of SVs linked with genes in shaping phenotypes, host–pathogen interactions, and environmental adaptation. These findings provide clues on the mechanism of adaptation, which need verification at the level of gene expression, proteome composition, biochemical mechanisms, and physiological effects. At the same time, we provide a valuable resource for future studies on cattle climate adaptation.

## Methods

### Samples collected for genome assembly, Nanopore LRS, and Illumina SRS

We sampled different tissues from 10 Hainan cattle in Hainan Province and from 10 Mongolian cattle in Alxa League, Inner Mongolia Autonomous Region, China, to build genome assemblies and/or LRS whole-genome sequencing. Genomic DNA was extracted from the tissues of the animals using the phenol/chloroform method.

For the two cattle whose genomes were assembled, testicular tissues were selected for long-read sequencing. Nanopore libraries were constructed using the Ligation Sequencing Kit (SQK-LSK109) and sequenced on PromethION (R9.4) flow cells. Base calling was performed using Guppy (v.5.1.13). Illumina paired-end libraries with an insert size of 350 bp were constructed using the NEB Next® Ultra™ DNA Library Prep Kit for Illumina (NEB, USA) following the manufacturer’s recommendations, and Hi-C libraries were prepared following the standard protocol described previously with certain modifications [58]. Both the Illumina paired-end and Hi-C libraries were sequenced on the Illumina platforms and 150 bp paired-end reads were generated.

The Illumina SRS data of 373 cattle from nine geographic regions (West Europe, North Europe, Central South Europe, Northeast Asia, Northwest China, South China, Southwest China, Central China, and South Asia) as well as 14 wild bovine species were generated in our study (*n* = 27) or retrieved from the sequence read archive of the National Center for Biotechnology Information (NCBI, Additional file 2: Table S13).

### Genome assembly and evaluation of genome quality

Raw Nanopore reads were corrected using NECAT [59] with the parameters “MIN_READ_LENGTH = 3000 and CNS_OUTPUT_COVERAGE = 60.” The same software was then used to assemble the contigs and further bridge the contigs using the corrected reads with the default parameters (Additional file 1: Fig. S1). The raw Nanopore reads were then mapped to the bridged contigs using Minimap2 [60] with the settings recommended for Oxford Nanopore sequencing data (-ax map-ont). Racon [61] was used to polish the bridged contigs with two iterations. Subsequently, the Illumina short reads were mapped back to the assemblies using the mem option of the Burrows–Wheeler Aligner (BWA) (v.0.7.13-r1126) [62]. We used SAMtools (v.1.3) [63] to sort the alignments and then applied Pilon [64] to polish the corresponding assemblies with two iterations.

Redundant sequences were removed from the assemblies by using HaploMerger2 [65]. The contigs were then anchored onto chromosomes by clean Hi-C reads through Juicer (v.1.5) [66] and 3D-DNA (v.201008) [67]. The assemblies were manually reviewed and adjusted using Juicebox Assembly Tools [68] (Additional file 1: Figs. S2 and S3). The completeness of the assemblies was assessed by the BUSCO (v.4.0) [18] analysis with the mammalia_odb9 database. The autosomal sequences of the two assemblies were consistent with the order of ARS_UCD1.2. *K*-mer completeness estimates were generated using the Merqury (v.1.0) pipeline [17].

### Genome annotation

Repetitive elements in Hainan_v1 and Mongolian_v1 were identified by their matches to Repbase (v.20140131) using RepeatMasker (v.4.0.5) (http://www.repeatmasker.org). To identify protein-coding genes in Mongolian_v1 and Hainan_v1, we used Liftoff (v.1.5.2) [69] and GffRead (v.0.12.1) [70] to generate gene annotations from ARS_UCD1.2. The annotation used for the transfer was NCBI GCF_002263795.1_ARS_UCD1.2_genomic.gff.

### Mapping rate comparison

We mapped the SRS data of Hainan (HN024) and Mongolian (NMG016) samples to Hainan_v1, Mongolian_v1, and ARS_UCD1.2 by using BWA-MEM. The summary of short-read mapping quality was generated by SAMtools. The mapping rate was calculated as the number of reads with a certain quality (MQ5/MQ10/MQ20/MQ30/MQ40/MQ50/MQ60) divided by the number of total reads.

### Variant calling evaluation

To assess the variant calling performance of the different references, the SRS data of 373 samples were aligned to each of the three assemblies (ARS_UCD1.2, Hainan_v1, and Mongolian_v1) using BWA-MEM (v.0.7.13-r1126) with default parameters, and the duplicated reads were removed using Picard Tools (http://broadinstitute.github.io/picard). The Genome Analysis Toolkit (GATK, v.3.8-1-0-gf15c1c3ef) was used to detect SNPs. The SNPs were called using the “HaplotypeCaller,” “GenotypeGVCFs,” and “SelectVariants” of GATK. After SNP calling, we used “VariantFiltration” to discard sequencing and alignment artifacts from the SNPs with the parameters “QD < 2.0, FS > 60.0, MQ < 40.0, MQRankSum <  − 12.5, ReadPosRankSum <  − 8.0 and SOR > 3.0,” and mean sequencing depth of variants (all individuals) “ < 1/3 × and > 3 × .”

### SVs discovery and genotyping

Two complementary approaches were applied to detect SVs in the Hainan and Mongolian genomes. First, Hainan_v1 and Mongolian_v1 were aligned using Minimap2 [60], and the resulting alignments were analyzed using Assemblytics [71] to call SVs. The minimum variant size was 10 bp. SV spanning gap regions were removed.

Second, LRS data from nine Mongolian cattle and nine Hainan cattle were aligned to Mongolian_v1. Mapping was performed using NGMLR (v.0.2.7) [24] with default parameters. SV calling was performed using CuteSV [22], SVIM [23], and Sniffles [24]. Five minimum supporting reads were needed for an SV. The minimum length of SVs was at least 50 bp in length. Specifically, CuteSV was run with the parameters “–max_cluster_bias_INS 100 –diff_ratio_merging_INS 0.3 –max_cluster_bias_DEL 100 –diff_ratio_merging_DEL 0.3 –min_support 5 –min_size 50 –genotype –report_readid –sample.” We set the following parameters for SVIM: “–min_sv_size 50 –insertion_sequences –sequence_alleles –read_names –sample.” Sniffles was run with the parameters “–num_reads_report -1 –report_seq –min_support 5 –min_length 50 –genotype” to remove positions marked as IMPRECISE for INFO or as UNRESOLVED for FILTER. SURVIVOR (v.1.0.7) [72] was used to merge the SVs supported by two or three calling methods with a maximum allowed pairwise distance of 1000 bp between breakpoints. After filtering out 4220 unreliable genotypes (all 0/0), we obtained a total of 108,231 LRS-based SVs.

The coordinates and features of all SVs called from both whole-genome alignment and LRS data were extracted and saved as bed files. The SVs called by these two methods were compared using BEDTools (v.2.25.0) [73] with a minimum reciprocal overlap of 80%. For insertions, we considered two SVs with breakpoints less than 80 bp to be the same SVs (Additional file 1: Fig. S20). The breakpoint setting refers to the parameters for humans [29]. We merged the SVs detected from these two methods for INSs and DELs and constructed a set of 123,898 nonredundant SVs.

These 123,898 nonredundant SVs were genotyped in 373 cattle that had been sequenced with SRS data. Reads from all SRS datasets were mapped to Mongolian_v1 using BWA-MEM (v.0.7.13-r1126) with default parameters. We used Paragraph (v.2.4a) [41] to genotype the combined SVs from the SRS data. BCFtools (v.1.9) [74] was used to combine the results for all genotypes. Then, we replaced all the unfiltered genotypes in Paragraph with missing genotypes (./.) and excluded SVs without any remaining nonreference genotypes. To obtain a high-quality dataset of genotyped SVs, we removed the SVs that were genotyped in less than 80% of cattle.

### Population genetic structure analysis

To construct the NJ phylogenetic tree and carry out the PCA using the SRS-based SVs, the two samples (HN024 and NMG016) employed for assembling Hainan_v1 and Mongolian_v1 were excluded. A matrix of pairwise genetic distances was calculated using PLINK [75] and used for construction and visualization of an unrooted NJ tree with MEGA (v.5.0) [76] and FigTree (v.1.4.3) (http://tree.bio.ed.ac.uk/software/figtree/). PCA was conducted using the smartPCA program in EIGENSOFT (v.5.0) [77].

### Detection of selection signals

To detect population-stratified SVs, we calculated the DI_SV_ [78] and *F*_ST_ distances between 93 northern and 142 southern Chinese cattle from eight and 13 breeds/populations, respectively. For DI_SV_, we first calculated the frequencies of individual SVs within populations using VCFtools (v.0.1.16) [79] with the –freq parameter, and the differences between the frequencies of southern and northern Chinese cattle were then used as the DI_SV_ values. For *F*_ST_-SVs, we employed the Weir and Cockerham estimator for *F*_ST_ estimates based on VCFtools (v.0.1.16) [79] to identify the population stratified SVs.

### Discovery of the introgressed SVs

We genotyped the SVs identified from this study (123,989 SVs) in three published high-coverage banteng genomes (Additional file 2: Table S13). Cattle from west Europe (*n* = 30), north Europe (*n* = 10), central south Europe (*n* = 15), South Asia (*n* = 5), and gaur (*n* = 2) were used as control populations for genotyping (Additional file 2: Table S13). We then investigated variants that were specific to Chinese indicine cattle and fixed in banteng genomes (allele frequency = 1) but absent from European taurine (allele frequency = 0) and South Asian indicine (allele frequency = 0) cattle. To reduce the effects of incomplete lineage sorting and complex evolutionary relationship across Asian bovine species [8], we also required the allele frequency of gaur to be zero. The –maf parameter of VCFtools (v.0.1.16) [79] was first used to filter the minor allele frequency of southern Chinese cattle. The same software with the –freq parameter was used to calculate the allelic frequency in different breeds/populations. We used *F*_ST_ (> 0.25) to investigate banteng-shared SVs that were highly stratified in Chinese indicine cattle. To verify these candidate introgressed SVs, we randomly selected SVs with different frequencies in banteng for IGV validation (v.2.2) [80]. RFMix (v.2.02) [81] was used to identify regions introgressed from banteng into Chinese indicine cattle using the SNPs. The introgressed fragments were defined by the following criteria: (1) fragments that shared ≥ 2 haplotypes in ≥ 2 samples and (2) ≥ 30 introgressed SNPs per fragment. We used IQTREE (v.1.6.6) [82] to construct a phylogenetic tree for the banteng introgressed regions. Modelfinder [83] was used to find the best model of a phylogenetic tree.

### Annotation of SVs

We extracted consistent sequences of INSs and DELs and compared them with consistent repeat sequences of mammals by RepeatMasker (v.4.0.5) to determine whether they were repetitive sequences. Functional regions of the SVs in the genomes were annotated using ANNOVAR [32]. KEGG and GO enrichment analyses were performed for SVs-linked genes in population stratification, and functional categories with adjusted *P* values lower than 0.05 were considered significantly enriched.

### SV validation

Visualization of detected SVs was performed using IGV (v.2.2) [80]. The target SVs were verified by PCR, agarose gel electrophoresis, and/or Sanger sequencing. For the exonic INS in the *SPN* gene, a pair of primers (forward: 5′-CGCCATGGGAGTCTTGAGAG-3′ and reverse: 5′-CTGCTTCTCCTCCTCTTCGG-3′) were used with standard PCR conditions. PCR was performed in 25-μL reaction volumes with 1 μL of genomic DNA (50 ng), 1 μL each of the forward and reverse primers (10 μM), and 22 μL of Golden Star T6 Super PCR mix (Beijing Tsingke Biotech Co., Beijing, China). PCR products were examined using 1.5% agarose-gel electrophoresis. The sizes of the amplified fragments were determined and used to infer the genotypes of the INS.

### Cell culture and treatment

RAW264.7 (ECACC 91062702) macrophage cell line was supplied by the Chinese Academy of Sciences Cell Bank and then cultured in high-glucose Dulbecco’s modified Eagle medium (DMEM) containing 10% fetal bovine serum (FBS) at 37 °C with 5% CO_2_. When the RAW264.7 cells reached 70% confluence, an appropriate amount of overexpression plasmids was transfected using Lipofectamine 3000 Reagent (Invitrogen, USA). The medium was replaced after the transfection for 12 h and culturing was continued for 12 h. Finally, the RAW264.7 cells were incubated together with the H37Ra strain of *M. tuberculosis* for 4 h and the medium was replaced for 12 h.

### Immunofluorescence

The H37Ra was first incubated with fluorescein isothiocyanate (FITC) for 1 h at 37 °C. Then, the H37Ra at different MOI values was incubated with the RAW264.7 cells for 4 h at 37 °C with 5% CO_2_. The infected RAW264.7 cells were fixed in 4% (v/v) paraformaldehyde in phosphate-buffered saline (PBS) at 4 °C for immunofluorescence analysis (Beyotime Institute of Biotechnology, Jiangsu, China). Thereafter, the RAW264.7 cells were incubated for 10 min at room temperature with DAPI. Finally, the RAW264.7 cells were viewed by the A1 confocal laser microscope (A1R, Nikon, Japan).

### RNA extraction and quantitative real-time PCR (qRT-PCR)

Total RNA from the RAW264.7 cells was isolated using the TRIzol Reagent (Invitrogen, Inc., CA, USA) and then reverse-transcribed into cDNA using the HiScript II 1st Strand cDNA Synthesis Kit (+ gDNA wiper) (Vazyme Biotech) according to the manufacturer’s instructions. qRT-PCR was performed using the EvaGreen qPCR Mastermix Kit (Vazyme AceQ® Universal SYBR® qPCR Master Mix) and CFX96 ™ real-time PCR detection system (Bio-Rad Laboratories, Inc., Hercules, USA). The relative levels of the target genes were calculated using the 2^−△△Ct^ method, and glyceraldehyde 3-phosphate dehydrogenase (*GAPDH*) was used as the reference gene. The data of qRT-PCR was analyzed using an unpaired *t*-test in the GraphPad Prism 6.0 software (GraphPad Software, San Diego, CA, USA, www.graphpad.com). For all analyses, *P* < 0.05 was considered statistically significant. Primer sequences are presented in Additional file 2: Table S17.

### ATAC-seq and ChIP-seq analysis

The ATAC-seq and ChIP-seq data were obtained from NCBI (Additional file 2: Table S18). After checking and trimming the adapters with Trim Galore (v.0.6.10) (https://github.com/FelixKrueger/TrimGalore), the ATAC-seq clean reads were mapped to Mongolian_v1 using Bowtie2 (v.2.4.5) [84]. BAM files were sorted using SAMtools (v.1.3) [63], and duplicate alignments were removed with Picard (v.2.20.2) (http://broadinstitute.github.io/picard). The peaks for individual replicates were called separately with MACS2 (v.2.2.7.1) [85] and then merged across three duplicates within the same tissue using BEDTools (v.2.25.0) [73]. The same pipeline was used for processing the ChIP-seq data.

### Supplementary Information

**Additional file 1: Fig. S1.** Flowchart of de novo assembly for the cattle genome. **Fig. S2.** Hi-C interaction heatmap of the Mongolian_v1 genome. **Fig. S3.** Hi-C interaction heatmap of the Hainan_v1 genome. **Fig. S4.** Alignment of the Mongolian_v1 assembly with the taurine cattle reference genome (ARS_UCD1.2). **Fig. S5.** Alignment of the Hainan_v1 assembly with the taurine cattle reference genome (ARS_UCD1.2). **Fig. S6.** Circos view of the assemblies of the Mongolian_v1 and Hainan_v1. **Fig. S7.** Distributions of read depths across the Hainan_v1 and Mongolian_v1 genomes. **Fig. S8.** Mapping rates (MQ) for the Illumina short reads from the HN024 and NMG016 samples against three different genomes (ARS_UCD1.2, Hainan_v1, and Mongolian_v1). **Fig. S9.** Candidate SVs on gene exons identified based on LRS data. **Fig. S10.** IGV screenshot of a 108-bp variation covering *SPN* in different reference genomes. **Fig. S11.** Genotyping of the 108-bp insertion of *SPN* using allele-specific PCR assay. **Fig. S12.** Alignment of complete SPN amino acid sequences in bovine species. **Fig. S13.** Distribution of insertions and deletions classified by intersected repeat elements. **Fig. S14.** The missing rate of each breed in SVs that failed for genotyping in at least 80% of the 373 cattle. **Fig. S15.** Principal components analysis (PCA) based on the SVs and SNPs of Illumina short reads in the 39 cattle breeds. **Fig. S16.** Model-based clustering was performed for SV of 39 cattle breeds using ADMIXTURE with the number of ancestry kinships (*k*) set to 2-6. **Fig. S17.** Neighbor-Joining trees constructed using SNPs in 39 cattle breeds with Illumina short reads mapped to the (A) ARS_UCD1.2, (B) Mongolian_v1, and (C) Hainan_v1 genomes. **Fig. S18.** An example of a 64-bp deletion (DEL) (BTA01:57,086,611-57,086,675, Mongolian_v1) located in the enhancer of *CD200* gene, which with high allele frequency difference (DI_SV_ = 0.635) and *F*_ST_-SV (0.588) between northern and southern Chinese cattle. **Fig. S19.** A 316-bp DEL of *DDX58* might be derived from banteng. **Fig. S20.** Breakpoint judgements for insertions.

**Additional file 2: Table S1.** Samples collected for genome assembly. **Table S2.** Quality evaluation of the assembled genomes using BUSCO (v3.0.2) software with the “mammalia_odb9” dataset. **Table S3.** Mapping ratio of Illumina reads to Hainan_v1 and Mongolian_v1 genomes. **Table S4.** Repeat annotation in the Mongolian_v1 genome. **Table S5.** Repeat annotation in the Hainan_v1 genome. **Table S6.** Polymorphism statistics. **Table S7.** SNP and Indel statistics of 373 Illumina data mapping to Hainan_v1, Mongolian_v1 and ARS_UCD1.2 genomes, respectively. **Table S8.** Genomic landscape of SVs (insertions and deletions) in the Hainan_v1 (Qry) and Mongolian_v1 (Ref) genomes. **Table S9.** Mapping ratio of Nanopore long reads to the Mongolian_v1 genome. **Table S10.** SVs statistics of different sets. **Table S11.** Population stratified structural variations (SVs) detected by LRS between Hainan and Mongolian cattle. **Table S12.** Distribution of LRS-based SV in different functional regions of the genome. **Table S13.** Summary of Illumina short-read sequencing data (mapping to Mongolian_v1 only chromosome scaffolds) used in this study. **Table S14.** Significantly enriched KEGG pathways and GO terms (adjusted *P* < 0.05) in genes affected by significant population stratification SVs. **Table S15.** Structural variations (SVs) that overlapped with ATAC peak. **Table S16.** North‒south stratified SVs that may be derived from banteng introgression. **Table S17.** The information of RT-PCR primers. **Table S18.** Summary of ATAC-seq and ChIP-seq data used in this study.

**Additional file 3.** Review history.

## Data Availability

The genome assemblies generated in this study are available in the NCBI under BioProject IDs of PRJNA810280 [86] and PRJNA810300 [87]. The Nanopore, Illumina HiSeq, and Hi-C data used for genome assembly can be accessed via the code of PRJNA823479 [88]. The source code of data analysis are publicly available under the GNU General Public License v.3.0 at GitHub [89] and Zenodo [90].
