# Supplementary material for: Structural variation and introgression from wild populations in East Asian cattle genomes confer adaptation to local environment
Source: Genome Biol. 2023 Sep 18;24:211. doi: 10.1186/s13059-023-03052-2 (PMC10507960; doi:10.1186/s13059-023-03052-2)
Supplement: Supplementary file 1 — Additional file 1: Fig. S1. Flowchart of de novo assembly for the cattle genome. Fig. S2. Hi-C interaction heatmap of the Mongolian_v1 genome. Fig. S3. Hi-C interaction heatmap of the Hainan_v1 genome. Fig. S4. Alignment of the Mongolian_v1 assembly with the taurine cattle reference genome (ARS_UCD1.2). Fig. S5. Alignment of the Hainan_v1 assembly with the taurine cattle reference genome (ARS_UCD1.2). Fig. S6. Circos view of the assemblies of the Mongolian_v1 and Hainan_v1. Fig. S7. Distributions of read depths across the Hainan_v1 and Mongolian_v1 genomes. Fig. S8. Mapping rates (MQ) for the Illumina short reads from the HN024 and NMG016 samples against three different genomes (ARS_UCD1.2, Hainan_v1, and Mongolian_v1). Fig. S9. Candidate SVs on gene exons identified based on LRS data. Fig. S10. IGV screenshot of a 108-bp variation covering SPN in different reference genomes. Fig. S11. Genotyping of the 108-bp insertion of SPN using allele-specific PCR assay. Fig. S12. Alignment of complete SPN amino acid sequences in bovine species. Fig. S13. Distribution of insertions and deletions classified by intersected repeat elements. Fig. S14. The missing rate of each breed in SVs that failed for genotyping in at least 80% of the 373 cattle. Fig. S15. Principal components analysis (PCA) based on the SVs and SNPs of Illumina short reads in the 39 cattle breeds. Fig. S16. Model-based clustering was performed for SV of 39 cattle breeds using ADMIXTURE with the number of ancestry kinships (k) set to 2-6. Fig. S17. Neighbor-Joining trees constructed using SNPs in 39 cattle breeds with Illumina short reads mapped to the (A) ARS_UCD1.2, (B) Mongolian_v1, and (C) Hainan_v1 genomes. Fig. S18. An example of a 64-bp deletion (DEL) (BTA01:57,086,611-57,086,675, Mongolian_v1) located in the enhancer of CD200 gene, which with high allele frequency difference (DISV = 0.635) and FST-SV (0.588) between northern and southern Chinese cattle. Fig. S19. A 316-bp DEL of DDX58 might be derived fr [file 13059_2023_3052_MOESM1_ESM.pdf]

Structural variation and introgression from wild populations in East Asian cattle genomes confer adaptation to local environment

Xiaoting Xia[1†], Fengwei Zhang[1†], Shuang Li[1†], Xiaoyu Luo[1†], Lixin Peng[2†], Zheng Dong[1†], Hubert Pausch[3], Alexander S. Leonard[3], Danang Crysnanto[3], Shikang Wang[1], Bin Tong[4], Johannes A. Lenstra[5], Jianlin Han[6,7], Fuyong Li[8], Tieshan Xu[9], Lihong Gu[10], Liangliang Jin[1], Ruihua Dang[1], Yongzhen Huang[1], Xianyong Lan[1], Gang Ren[1], Yu Wang[1], Yuanpeng Gao[11], Zhijie Ma[12], Haijian Cheng[1,13], Yun Ma[14], Hong Chen[1], Weijun Pang[1*], Chuzhao Lei[1*], Ningbo Chen[1*]

[1] Key Laboratory of Animal Genetics, Breeding and Reproduction of Shaanxi Province, College of Animal Science and Technology, Northwest A&F University, Yangling, China;
[2] National Engineering Research Center for Non-food Biorefinery, Guangxi Academy of Sciences, 98 Daling Road, Nanning, China;
[3] Animal Genomics, ETH Zurich, Universitaetstrasse 2, 8006 Zurich, Switzerland;
[4] The State Key Laboratory of Reproductive Regulation and Breeding of Grassland Livestock, School of Life Sciences, Inner Mongolia University, Hohhot, China;
[5] Faculty of Veterinary Medicine, Utrecht University, Utrecht, The Netherlands;
[6] Livestock Genetics Program, International Livestock Research Institute (ILRI), Nairobi, Kenya;
[7] CAAS-ILRI Joint Laboratory on Livestock and Forage Genetic Resources, Institute of Animal Science, Chinese Academy of Agriculture Sciences (CAAS), Beijing, China;
[8] Department of Infectious Diseases and Public Health, Jockey Club College of Veterinary Medicine and Life Sciences, City University of Hong Kong, Kowloon, Hong Kong SAR, China;
[9] Tropical Crops Genetic Resources Institute, Chinese Academy of Tropical Agricultural Sciences, Haikou China;
[10] Institute of Animal Science & Veterinary Medicine, Hainan Academy of Agricultural Sciences, Haikou China;
[11] College of Veterinary Medicine, Northwest A&F University, Yangling, China;
[12] Qinghai Academy of Animal Science and Veterinary Medicine, Qinghai University, Xining, China;
[13] Institute of Animal Science and Veterinary Medicine, Shandong Academy of Agricultural Sciences, Shandong Key Lab of Animal Disease Control and Breeding, Jinan, China;
[14] Key Laboratory of Ruminant Molecular and Cellular Breeding of Ningxia Hui Autonomous Region, School of Agriculture, Ningxia University, Yinchuan, China.
†Xiaoting Xia, Fengwei Zhang, Shuang Li, Xiaoyu Luo, Lixin Peng and Zheng Dong contributed equally to this work.
*Correspondence: pwj1226@nwafu.edu.cn; leichuzhao1118@nwafu.edu.cn; ningbochen@nwafu.edu.cn

Contents

Fig. S1. Flowchart of de novo assembly for the cattle genome.

Fig. S2. Hi-C interaction heatmap of the Mongolian_v1 genome.

Fig. S3. Hi-C interaction heatmap of the Hainan_v1 genome.

Fig. S4. Alignment of the Mongolian_v1 assembly with the taurine cattle reference genome (ARS_UCD1.2).

Fig. S5. Alignment of the Hainan_v1 assembly with the taurine cattle reference genome (ARS_UCD1.2).

Fig. S6. Circos view of the assemblies of the Mongolian_v1 and Hainan_v1.

Fig. S7. Distributions of read depths across the Hainan_v1 and Mongolian_v1 genomes.

Fig. S8. Mapping rates (MQ) for the Illumina short reads from the HN024 and NMG016 samples against three different genomes (ARS_UCD1.2, Hainan_v1, and Mongolian_v1).

Fig. S9. Candidate SVs on gene exons identified based on long-read sequencing data.

Fig. S10. IGV screenshot of a 108-bp variation covering *SPN* in different reference genomes.

Fig. S11. Genotyping of the 108-bp insertion of *SPN* using allele-specific PCR assay.

Fig. S12. Alignment of complete SPN amino acid sequences in bovine species.

Fig. S13. Distribution of insertions and deletions classified by intersected repeat elements.

Fig. S14. The missing rate of each breed in SVs that failed for genotyping in at least 80% of the 373 cattle.

Fig. S15. Principal components analysis (PCA) based on the SVs and SNPs of Illumina short reads in the 39 cattle breeds.

Fig. S16. Model-based clustering was performed for SV of 39 cattle breeds using ADMIXTURE with the number of ancestry kinships (*k*) set to 2-6.

Fig. S17. Neighbor-Joining trees constructed using SNPs in 39 cattle breeds with Illumina short reads mapped to the (A) ARS_UCD1.2, (B) Mongolian_v1, and (C) Hainan_v1 genomes.

Fig. S18. An example of a 64-bp deletion (DEL) (BTA01:57,086,611-57,086,675, Mongolian_v1) located in the enhancer of *CD200* gene, which with high allele frequency difference (DI_{SV} = 0.635) and F_{ST}-SV (0.588) between northern and southern Chinese cattle.

Fig. S19. A 316-bp DEL of *DDX58* might be derived from banteng.

Fig. S20. Breakpoint judgements for insertions.

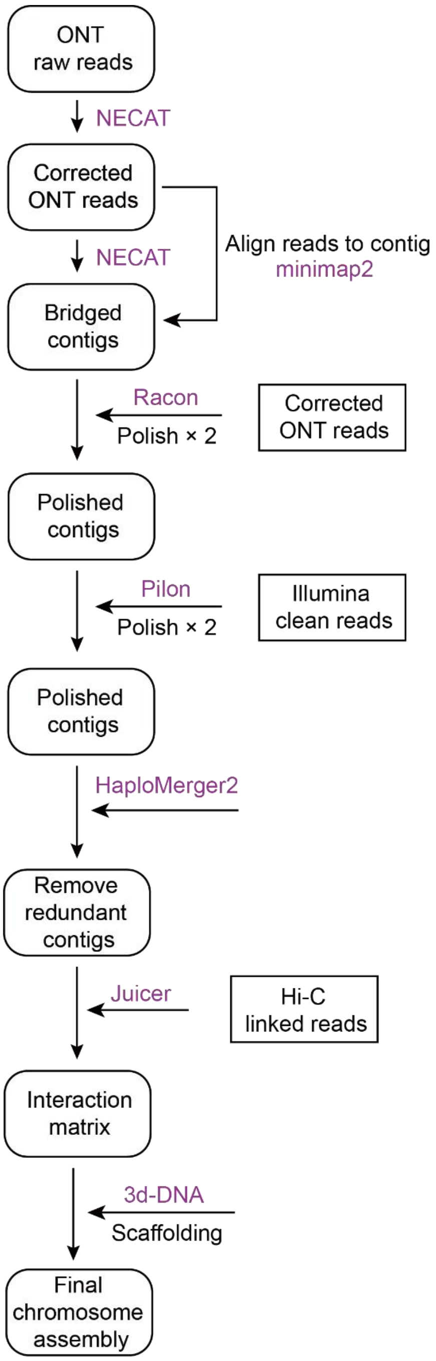

Fig. S1. Flowchart of *de novo* assembly for the cattle genome.

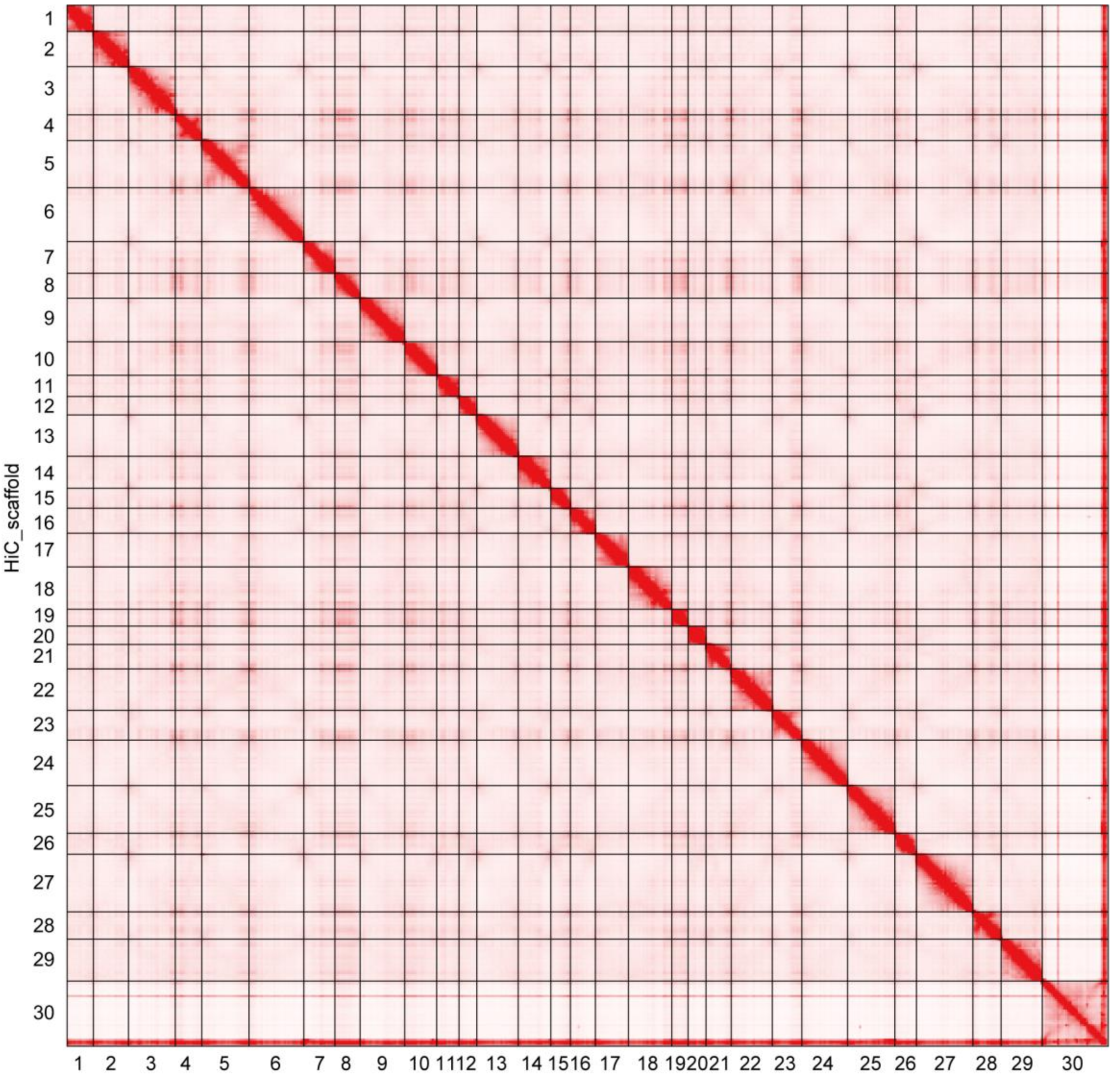

Fig. S2. Hi-C interaction heatmap of the Mongolian_v1 genome.

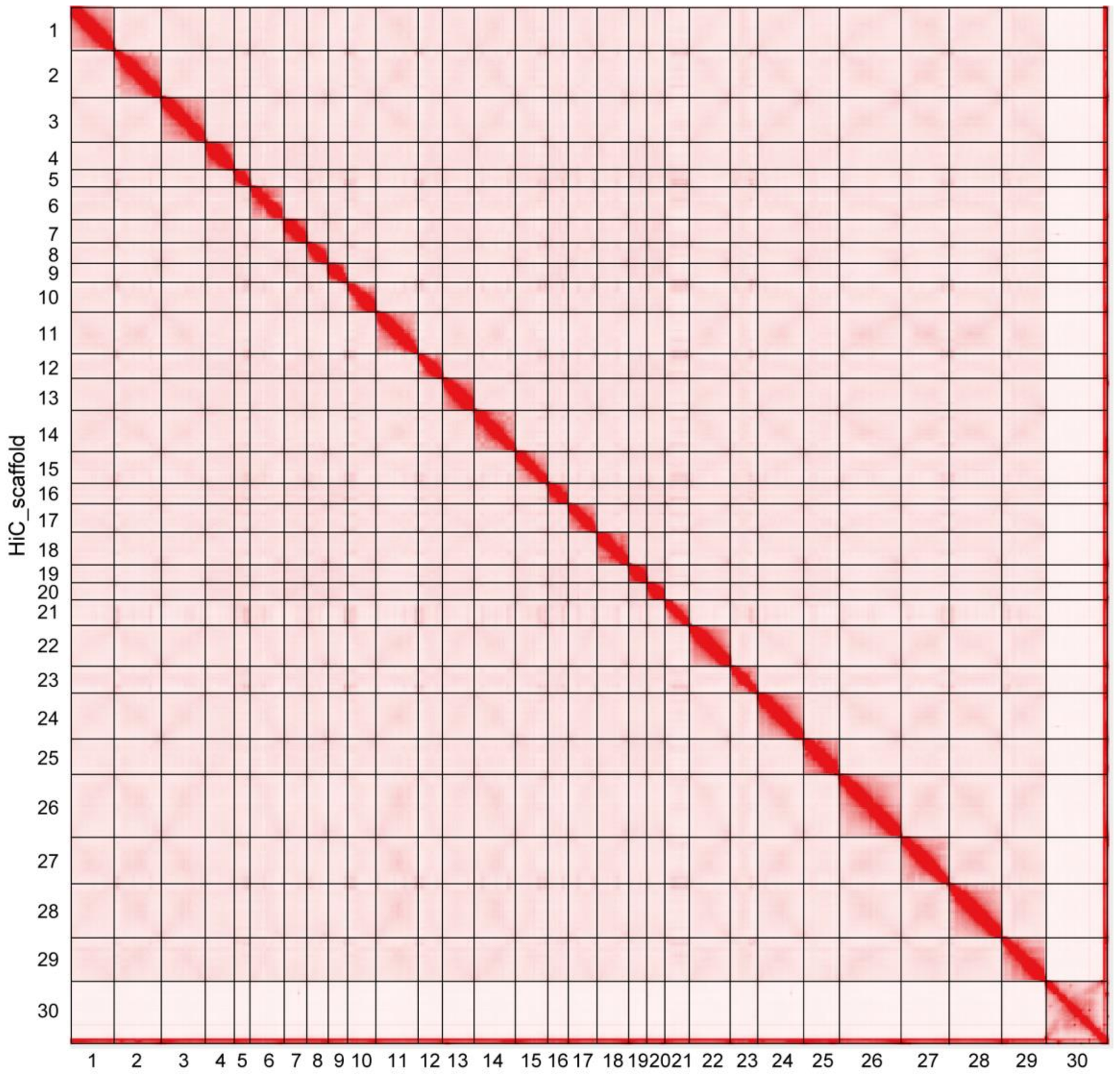

Fig. S3. Hi-C interaction heatmap of the Hainan_v1 genome.

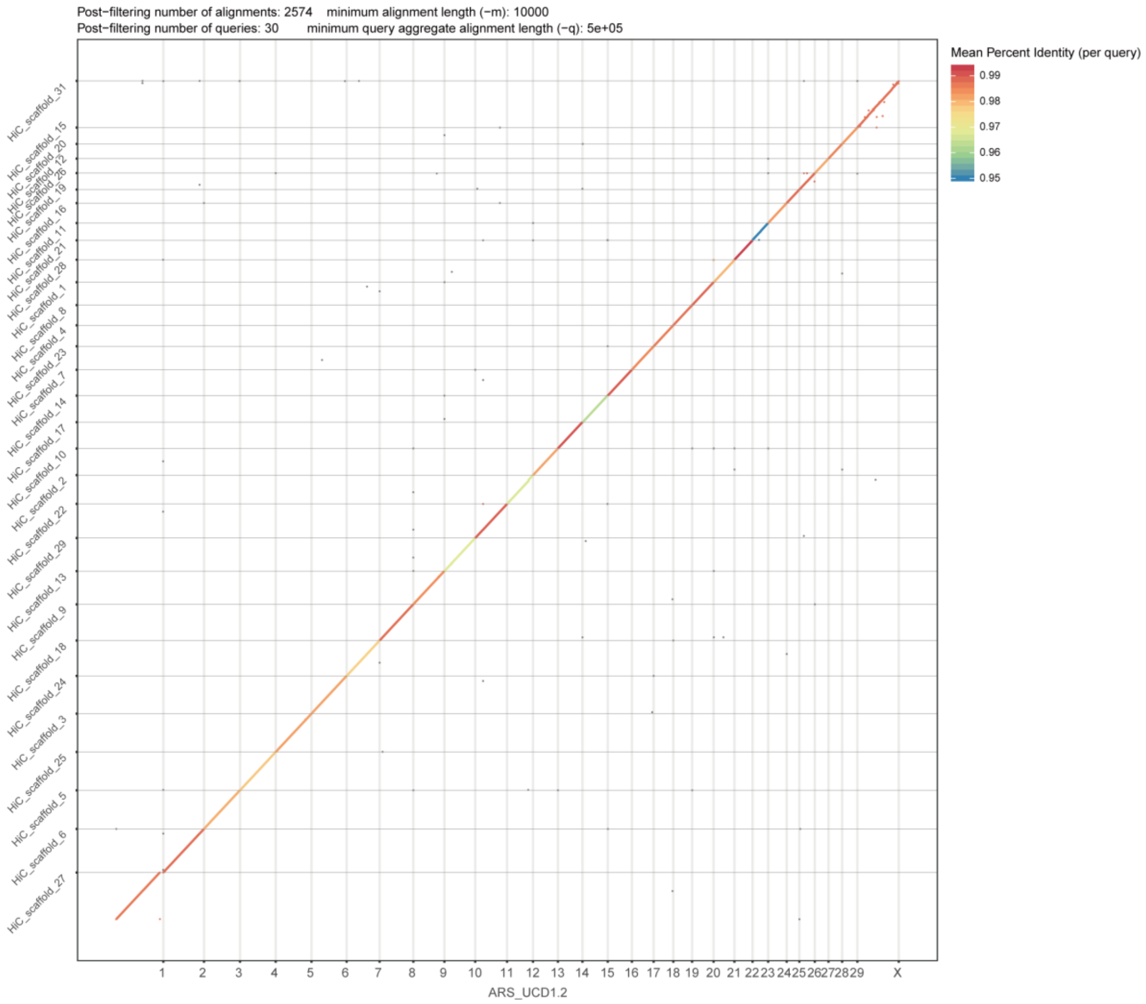

Fig. S4. Alignment of the Mongolian_v1 assembly with the taurine cattle reference genome (ARS_UCD1.2).

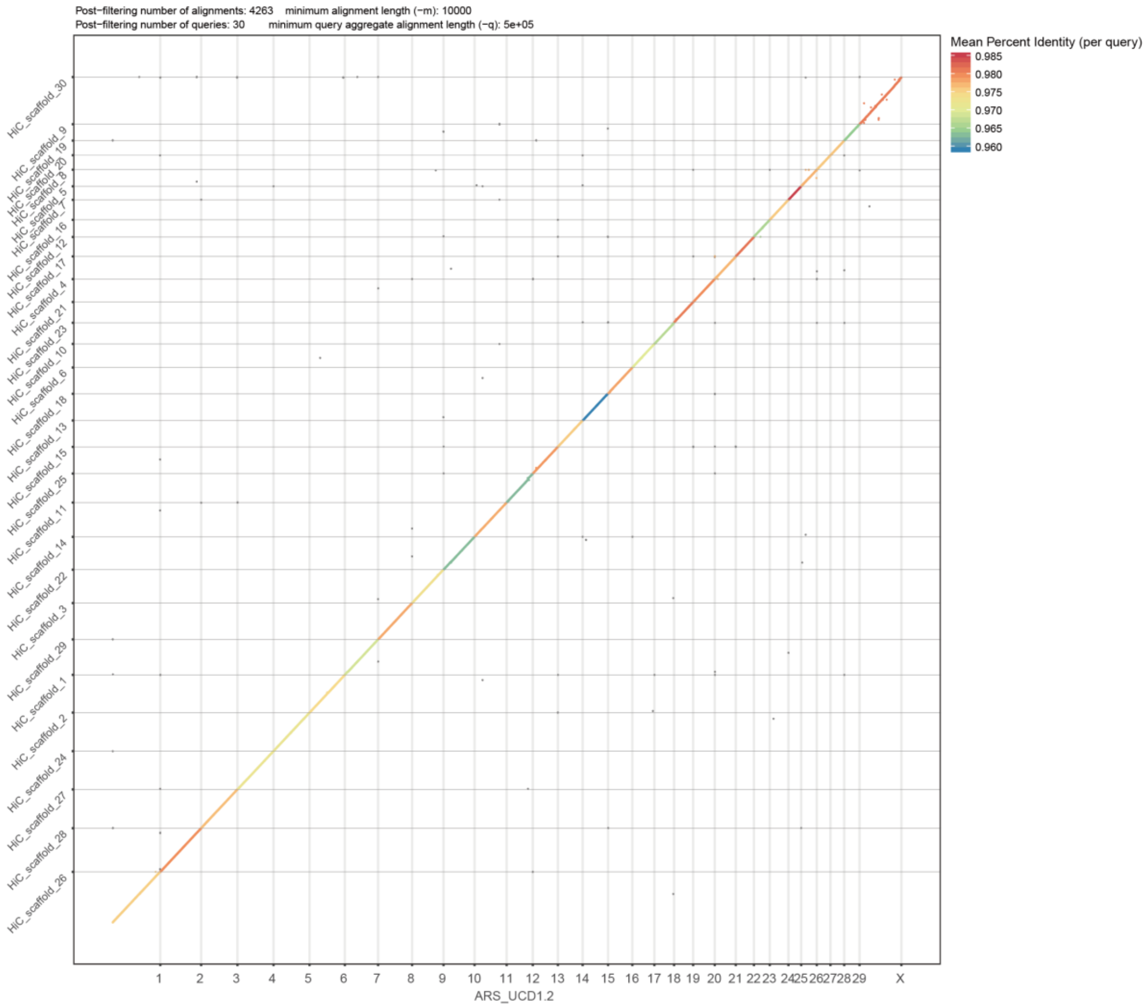

Fig. S5. Alignment of the Hainan_v1 assembly with the taurine cattle reference genome (ARS_UCD1.2).

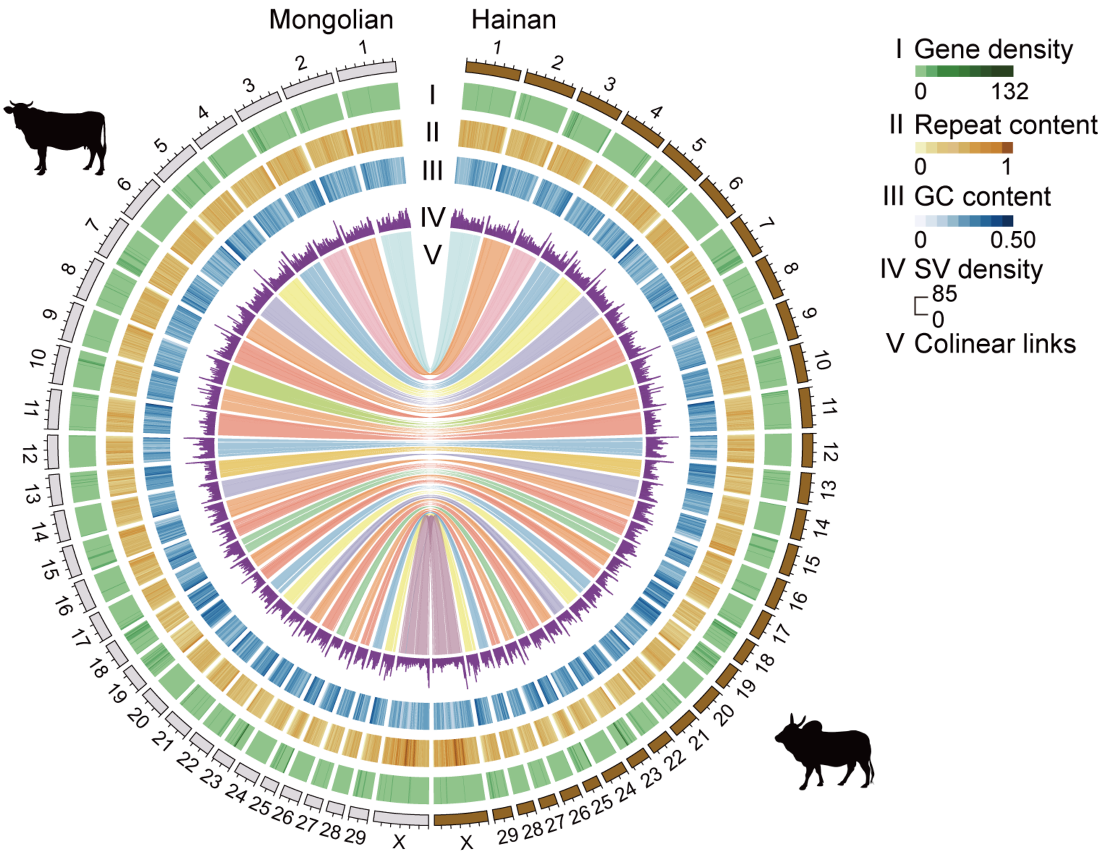

Fig. S6. Circos view of the assemblies of the Mongolian_v1 and Hainan_v1. Track I is for gene density, track II repeat content, track III GC content, track IV SV density, and track V colinear links.

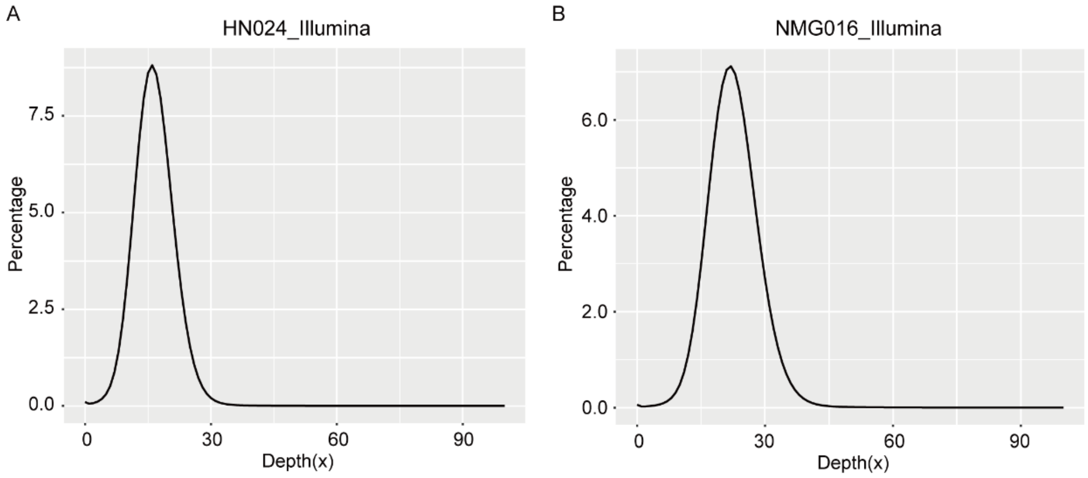

Fig. S7. Distributions of read depths across the Hainan_v1 and Mongolian_v1 genomes. Illumina short reads used for genome assembly were mapped back to their own genomes. Average mapping depths of the short reads for the Hainan and Mongolian cattle were 18.091× (A) and 25.593 × (B), respectively.

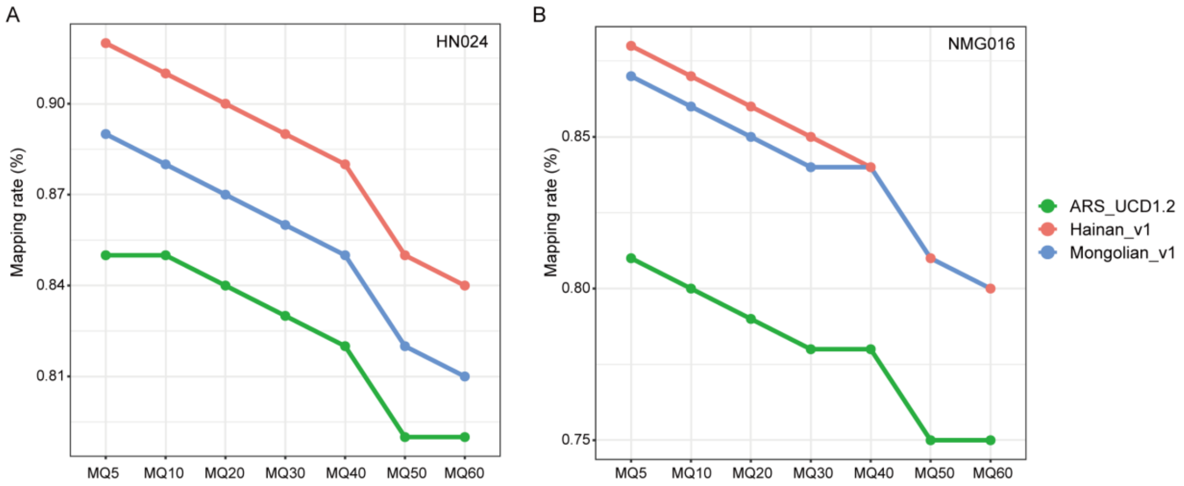

Fig. S8. Mapping rates (MQ) for Illumina short reads from the HN024 and NMG016 samples against three different assemblies (ARS_UCD1.2, Hainan_v1, and Mongolian_v1). The horizontal axis represents different MQ cutoffs.

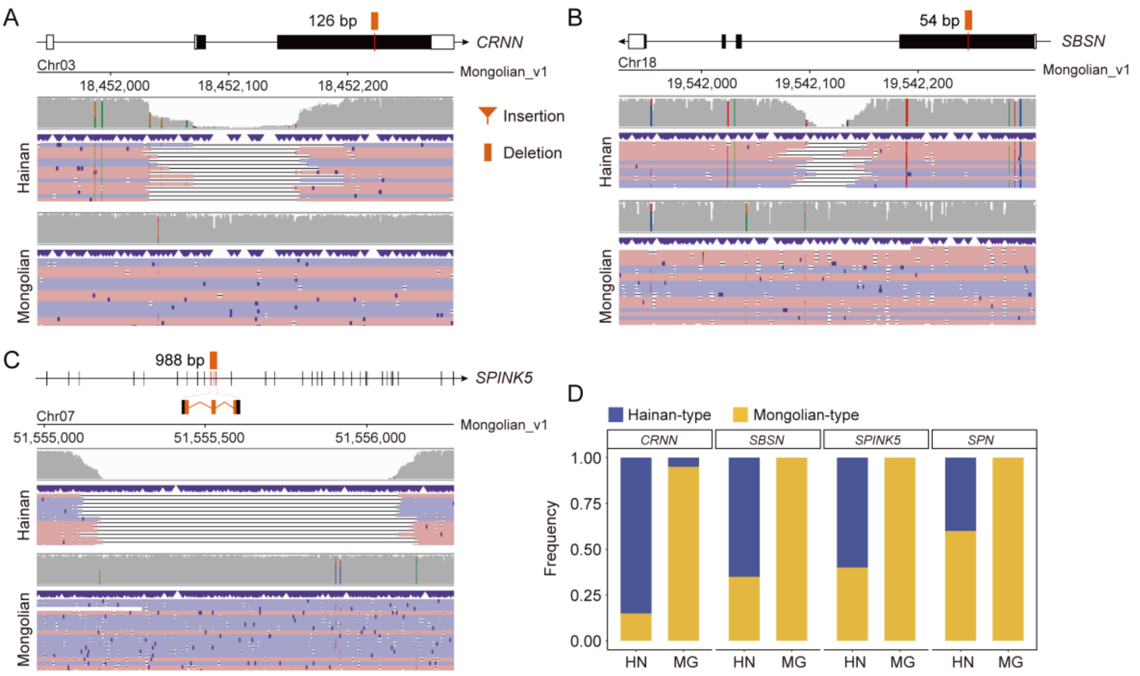

Fig. S9. Candidate SVs on gene exons identified based on long-read sequencing data. (A) A 126-bp DEL in the third exon of *CRNN*. (B) A 54-bp DEL in exon 1 of *SBSN*. (C) A 988-bp DEL in exons 10 to 12 of *SPINK5*. (D) Allele frequencies of four candidate SVs in Hainan and Mongolian cattle. HN, Hainan breed; MG, Mongolian breed.

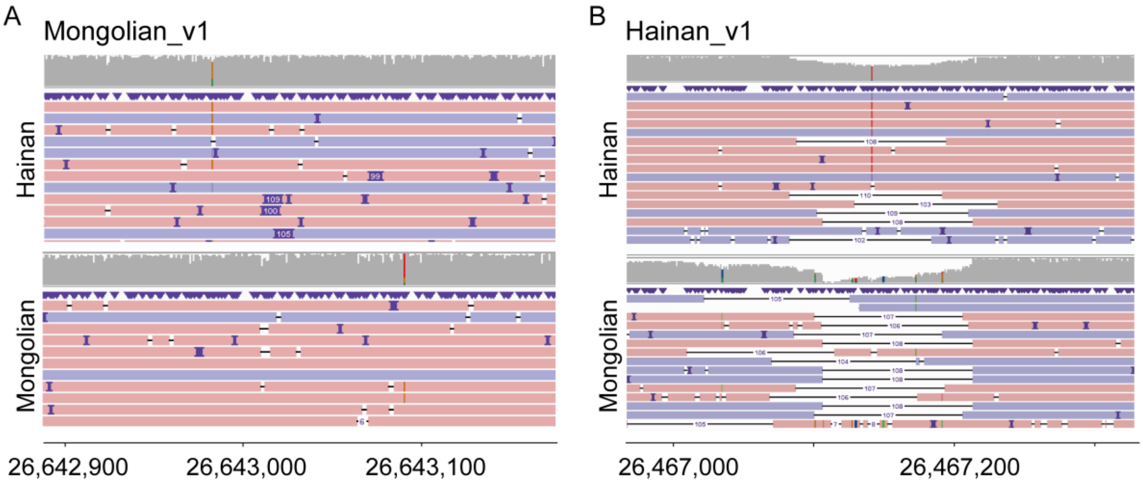

Fig. S10. IGV screenshot of a 108-bp variation covering *SPN* in different reference genomes. (A) The mapping of LRS data using Mongolian_v1 as reference genome. (B) The mapping of LRS data using Hainan_v1 as reference genome.

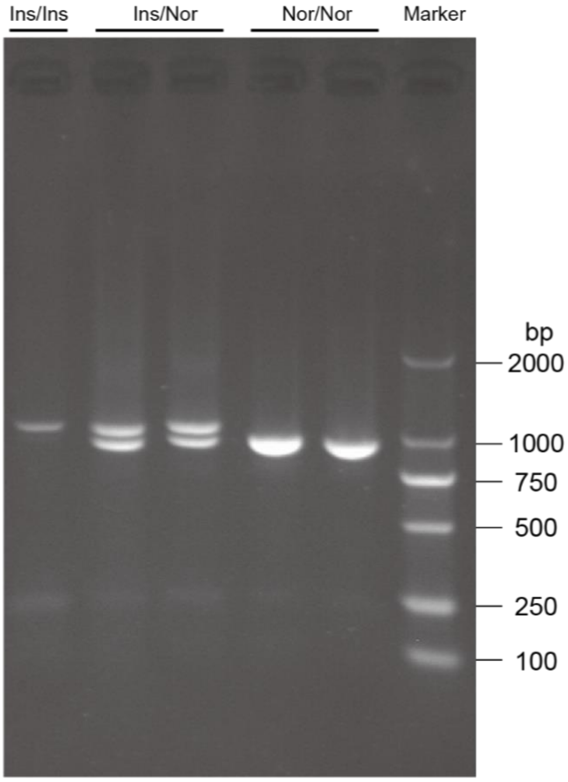

Fig. S11. Genotyping of the 108-bp insertion of *SPN* using allele-specific PCR assay. PCR products of the wild allele (Normal; Nor) and mutant allele (Insertion; Ins) in five samples are separated using agarose gel electrophoresis.

```
#Hainan                    ---------- ------MGAE NLTEVSSRSP PLWTSKTKIA SLSSVSETLT FNSVTTNLTT NEVSKMSDNP EHQTLPPSSI  [ 80]
#Bos_indicus_x_Bos_taurus  ------MILL LLLFGSMGAE NVTEVSSRSS PLWTSKTTIA SLSSVSETLT FNSVTTSLTT NEVSKMSDNP EHQTLPPSSI  [ 80]
#taurine                   ---------- ------MGAE NVTEVSSRSS PLWTSKTTIA SLSSVSETLT FNSVTTSLTT NEVSKMSDNP EHQTLPPSSI  [ 80]
#bison                     ------MILL LLLFGSMGAE NLTEVSSRSS PLWTSKTTIA SLSSVSETLT FNSVTTSLTT NEVSKMSDNP EHQTLPPSSI  [ 80]
#wisent                    ---------- ------MGAE NLTEVSSRSS PLWTSKTTIA SLSSVSETLT FNSVTTSLTT NEVSKMSDNP EHQTLPPSSI  [ 80]
#yak                       ---------- ------MGAE NLTEVSSRSS PLWTSKTTIA SLSSVSETLT FNSVTTSLTT NEVSKMSDNP EHQTLPPSSI  [ 80]
#buffalo                   MPVALEMILL LLLFGSMGAE NLTEVSPRSS PLWTSKATIA SLSSVSETLP FNLVTTSLTT NKVSKMSDNP EHQILPPSSI  [ 80]

#Hainan                    PYIADVVSSP ETSPTASRGS PVSESTISKE DSSKKSIKLM KTPDATSTPG VSVMKPTGFP TMTSKTMATS PLETS-----  [160]
#Bos_indicus_x_Bos_taurus  PYIADVVSSP ETSPTASRGS PVSESTISKE DSSKKSIKLM KTPDATSTPG VSVMKPTGFP TMTSETMATS PLETS-----  [160]
#taurine                   PYIADVVSSP ETSPTASRGS PVSESTISKE DSSKKSIKLM KTPDATSTPG VSVMKPTGFP TMTSKTMATS PLETS-----  [160]
#bison                     PYIADVVSSP ETSPTASRGS PVSESTISEE DSIKKSIKLM EIPDATSTPG VSVMKPTGFP TMTSETMATN PLETS-----  [160]
#wisent                    PYIADVVSSP ETSPTASRGS PVSESTISKE DSIKKSIKLM EIPDATSTPG VSVMKPTGFP TMTSETMATN PLETS-----  [160]
#yak                       PYIADVVSSP ETSPTASRGS PVSESTISKE DSIKKSIKLM EIPDATSTPG VSVMKPTGFP TMTSETMATN PLETS-----  [160]
#buffalo                   PYIANVVSSP ETSPTASRGS PVSESTISQE DS-IKSIKPM EISDAPSTTG VSVMKPTGFP TMTSETMATS PLETSNGTSG  [160]

#Hainan                    -------SAT SKVLLTMATS SLEISGGTSR PPVSMATSSL DTSSGTSEVL LTMATSSLDI SGGTSRPPVS MAMSSLDTSS  [240]
#Bos_indicus_x_Bos_taurus  -------SAT SKVLLTMATS SLEISG---- ---------- ---------- ---------- --GTSRPPVS MATSSLDTSS  [240]
#taurine                   -------SAT SKVLLTMATS SLEISG---- ---------- ---------- ---------- --GTSRPPVS MAMSSLDTSS  [240]
#bison                     -------SAT SKVLLTMATS ---------- ---------- ---------- ---------- ---------- ----------  [240]
#wisent                    -------SAT SKVLLTMATS SLEISG---- ---------- ---------- ---------- --GTSRPPVS MATSSLDTSS  [240]
#yak                       -------SAT SKVLLTMATS SLEISG---- ---------- ---------- ---------- --GTSRPPVS MATSSLDTSS  [240]
#buffalo                   TSPLETSSAT SKVLLTMATN SLEISG---- ---------- ---------- ---------- --GTSRPPVT MATSSLETAS  [240]

#Hainan                    ------GTSE VLLTMATSSL DISGGTSRPP VSMATSSLDT SSGTSEVLLT MATSSLDISG GTSRPPVTMA TSSLETSSGT  [320]
#Bos_indicus_x_Bos_taurus  ------GTSE VLLTMATSSL DISGGTSRPP VSMAMSSLDT SSGTSEVLLT MATSSLEISG GTSRPPVSMA TSSLDTSSGT  [320]
#taurine                   ------GTSE VLLTMATSSL DISGGTSRPP VSMATSSLDT SSGTSEVLLT MATSSLDISG GTSRPPVTMA TSSLETSSGT  [320]
#bison                     ---------- --------SL DISGGTSRPP VSMATSSLDT SSGTSEVLLT MATSSLDISG GTSRPPVTMA TSSLETSSGT  [320]
#wisent                    ------GTSE VLLTMATSSL DISGGTSRPP VSMATSSLDT SSGTSEVLLT MATSSLDISG GTSRPPVTMA TSSLETSSGT  [320]
#yak                       GTSEVLGTSE VLLTMATSSL DISGGTSRPP VSMATSSLDT SSGTSEVLLT MATSSLDISG GTSRPPVTMA TSSLETSSGT  [320]
#buffalo                   ------GTSK VLLTMATSSL ELSGGTSRPP VTMATSSLET ASGTSKVLLT MATSSLELSG GTSRPPVTMA TSSLETSSGT  [320]

#Hainan                    REPLVTTATS SVKTISMTSG SPVIMKTSSP KTSKG-SGLL VTMPATSLKT PMGTTGSTGH EGTTFSPNTS TNVSRRDKLI  [400]
#Bos_indicus_x_Bos_taurus  REPLVTTATS SVKTISMTSG SPVIMKTSSP KTSKG-SGLL VTTPATSLKT PMATTGSTGH EVTTFSPNTS TNVSRRDKLI  [400]
#taurine                   REPLVTTATS SVKTISMTSG SPVIMKTSSP KTSKG-SGLL VTTPATSLKT PMATTGSTGH EVTTFSPNTS TNVSRRDKLI  [400]
#bison                     REPLVTTATS SVKTISMTSG SPVIMKTSSP KTSKG-SGLM VTMPATSLKT PMGTTGSTGH EVTTFSPNTS TNISRRDKLI  [400]
#wisent                    REPLVTTATS SVKTISMTSG SPVIMKTSSP KTSKG-SGLL VTMPATSLKT PMGTTGSTGH EVTTFSPNTS TNVSRRDKLI  [400]
#yak                       REPLVTTATS SVKTISMTSG SPVIMKTSSP KTSKG-SGLL VTMPATSLKT PMGTTGSTGH EVTTFSPNTS TNVSRRDKLI  [400]
#buffalo                   REPLVTTATS SVKTISMTSG SPVIMKTSSP KTSKGTSGLL STMPATSLKT PMGTSGSTSH EVTTFSPNTS TNISRRDKLI  [400]

#Hainan                    PPQGTKGTLL VAVLVALLVV VVLVALILLW LRRQKRKTGV LTLGGGGKRN GVVDAWAGVA RVPDEEAMTA TEGASRGNND  [480]
#Bos_indicus_x_Bos_taurus  PPQGTKGTLL VAVLVALLVV VVLVALILLW LRRQKRKTGV LTLGGGGKRN GVVDAWAGVA RVPDEEAMTA TEGASRGNND  [480]
#taurine                   PPQGTKGTLL VAVLVALLVV VVLVALILLW LRRQKRKTGV LTLGGGGKRN GVVDAWAGVA RVPDEEAMTA TEGASRGNND  [480]
#bison                     PPQGTKGTLL VAVLVALLVV VVLVALILLW LRRQKRKTGV LTLGGGGKRN GVVDAWAGVA RVPDEEAMTA TEGASRGNND  [480]
#wisent                    PPQGTKGTLL VAVLVALLVV VVLVALILLW LRRQKRKTGV LTLGGGGKRN GVVDAWAGVA QVPDEEAMTA TEGASRGNND  [480]
#yak                       PPQGTKGTLL VAVLVALLVV VVLVALILLW LRRQKRKTGV LTLGGGGKRN GVVDAWAGVA RVPDEEAMTA TEGASRGNND  [480]
#buffalo                   PAQGTKGTLL VAVLVALLVV VVLVALILLW LRRQKRKTGV LTLGSSWKRN GAVDAWAGVA QVPDEEAMTA TEGASGGNND  [480]

#Hainan                    SDGPHREGSG QRPTLTTFFG RRKSRQGSMA LEELKAGPAS SLKGEEEPLV CNEDEGAEAP TSNGPEAREV KTP [553]
#Bos_indicus_x_Bos_taurus  SDGPHREGSG QRPTLTTFFG RRKSRQGSMA LEELKAGPAS SLTGEEEPLV CNEDEGAEAP TSNGPEAREV KTP [553]
#taurine                   SDGPHREGSG QRPTLTTFFG RRKSRQGSMA LEELKAGPAS SLTGEEEPLV CNEDEGAEAP TSNGPEAREV KTP [553]
#bison                     SDGPHREGSG QRPTLTTFFG RRKSRQGSMA LEELKAGPAS SLKGEEEPLV CNEDEGAEAP TSNGPEAREV KTP [553]
#wisent                    SDGPHREGSG QRPTLTTFFG RRKSRQGSMA LEELKAGPAS SLKGEEEPLV CNEDEGAEAP TSNGPEAREV KTP [553]
#yak                       SDGPHREGSG QRPTLTTFFG RRKSRQGSMA LEELKAGPAS SLKGEEEPLV CNEDEGAEAP TSNGPEAREV KTP [553]
#buffalo                   SDGPHREGSG QRPTLTTFFG RRKSRQGSMA LEELKAGPAS SLKGEEEPLV CNEDEGAEAP TSNGPEAREV KTP [553]
```

Fig. S12. Alignment of complete SPN amino acid sequences in bovine species.

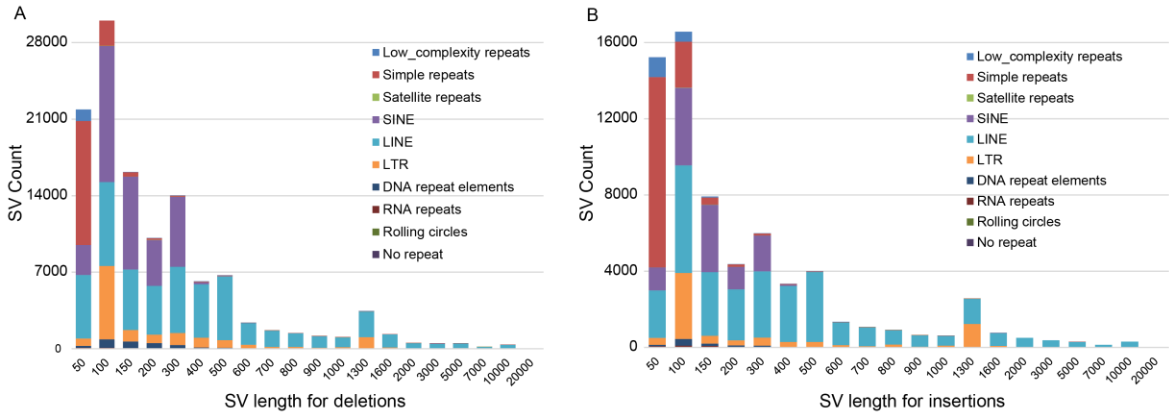

Fig. S13. Distribution of insertions and deletions classified by intersected repeat elements.

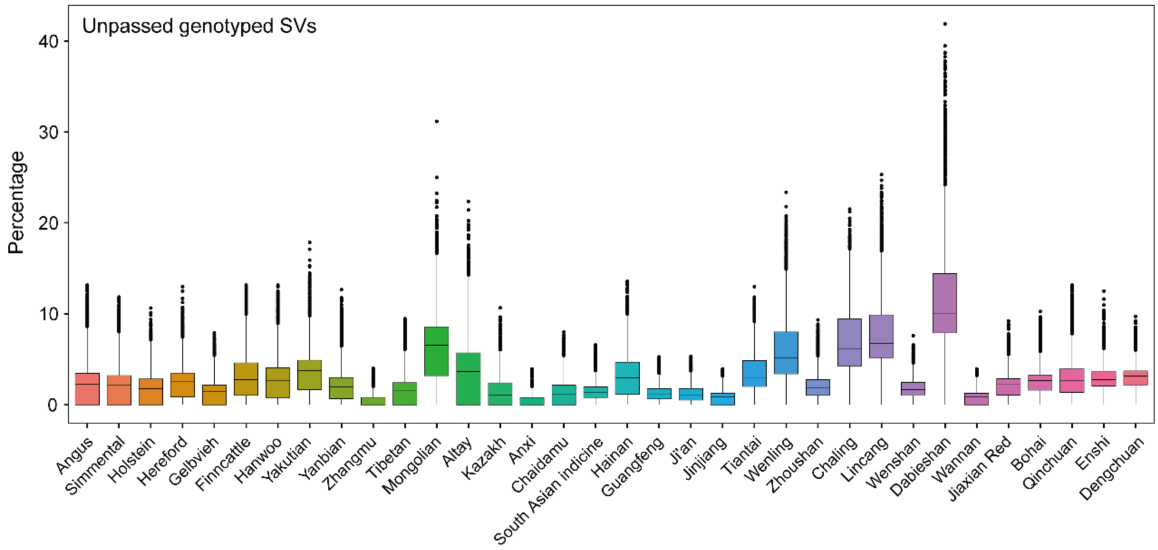

Fig. S14. The missing rate of each breed in SVs that failed for genotyping in at least 80% of the 373 cattle. The “South Asian indicine” includes Gir, Nelore, Tharparkar, Sahiwal, and Hariana breeds.

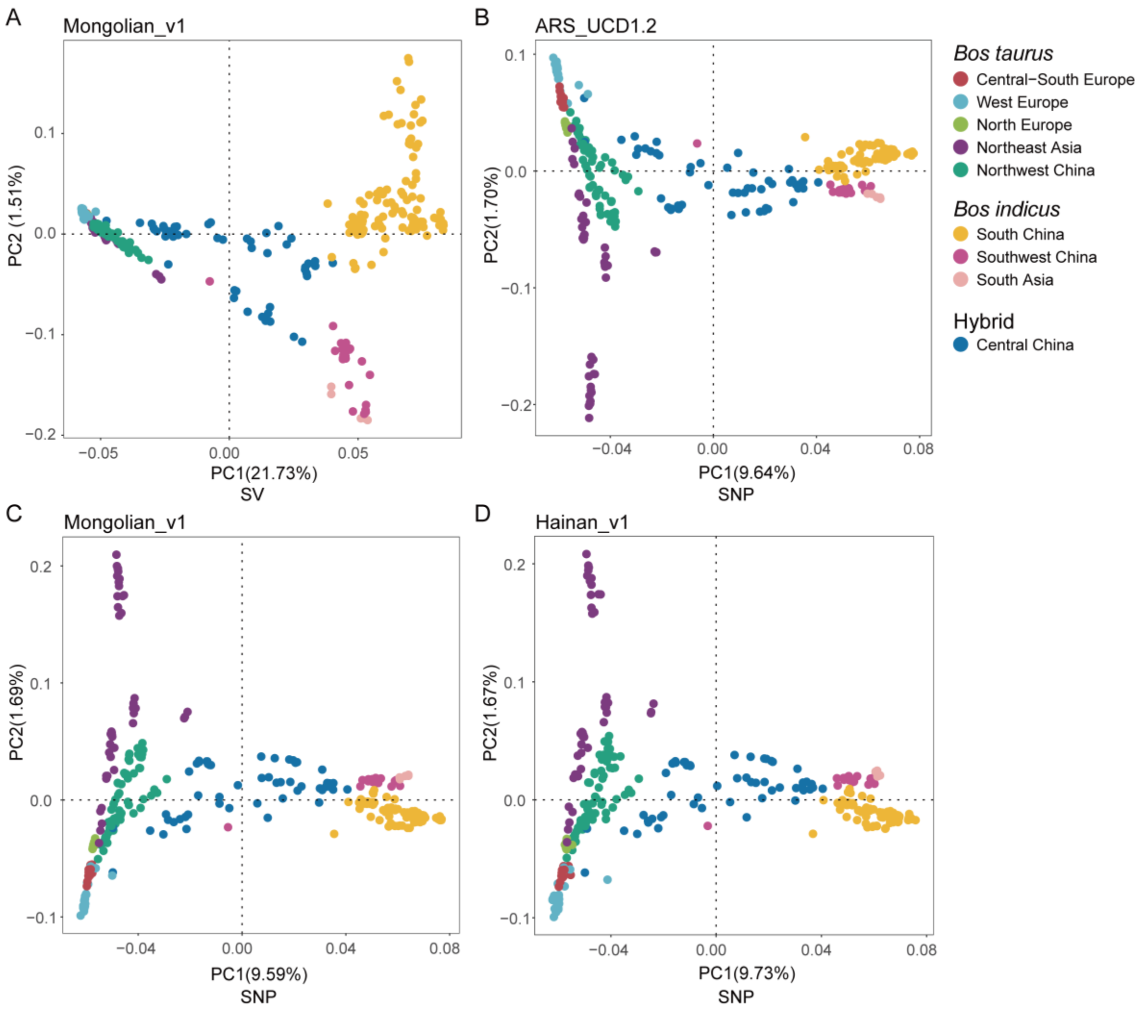

Fig. S15. Principal components analysis (PCA) based on the SVs and SNPs of Illumina short reads in the 39 cattle breeds. PCA based on the genotypes of SVs (A) and SNPs mapped against the ARS_UCD1.2 (B), Mongolian_v1 (C) or Hainan_v1 (D) assembly.

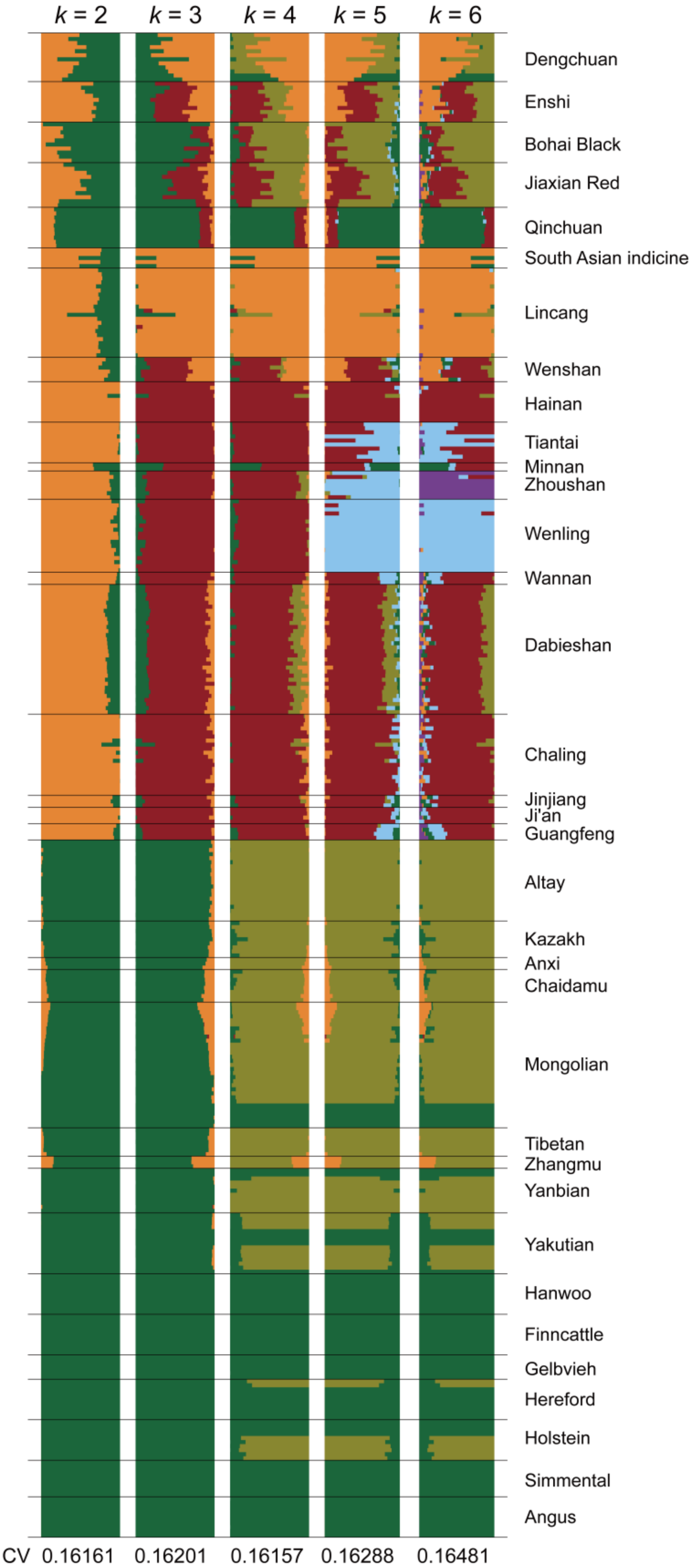

Fig. S16. Model-based clustering was performed for SV of 39 cattle breeds using ADMIXTURE with the number of ancestry kinships (*k*) set to 2-6.

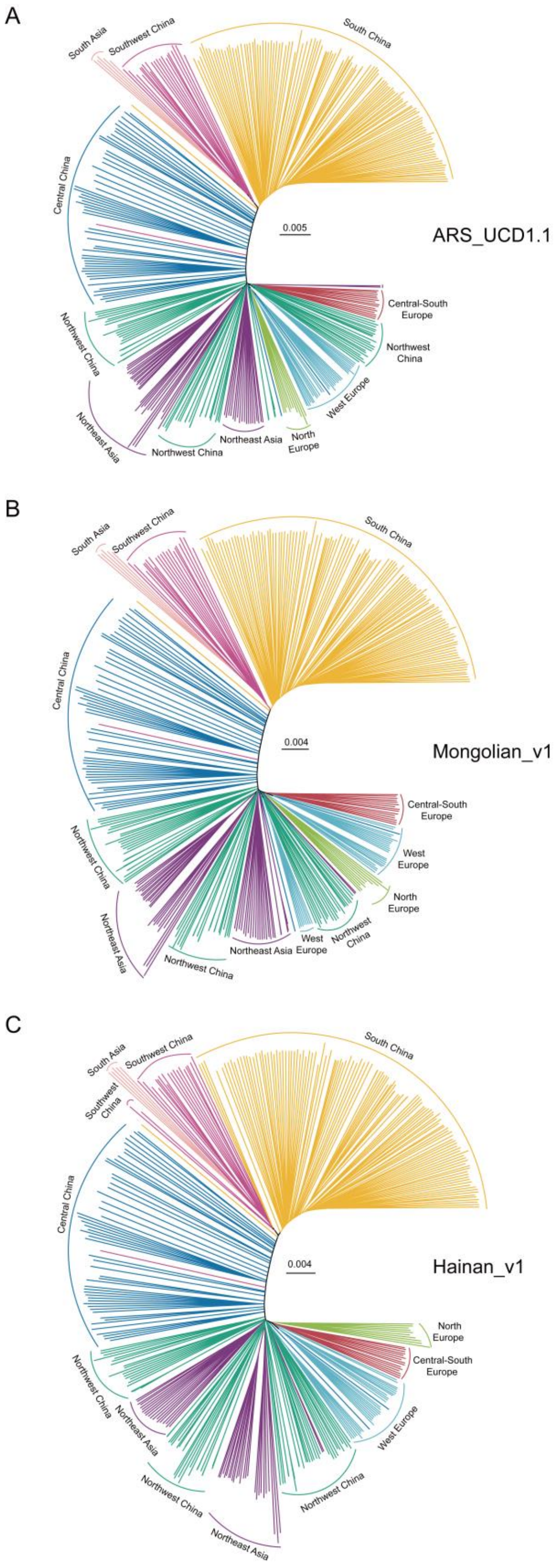

Fig. S17. Neighbor-Joining trees constructed using SNPs in 39 cattle breeds with Illumina short reads mapped to the (A) ARS_UCD1.2, (B) Mongolian_v1, and (C) Hainan_v1 genomes.

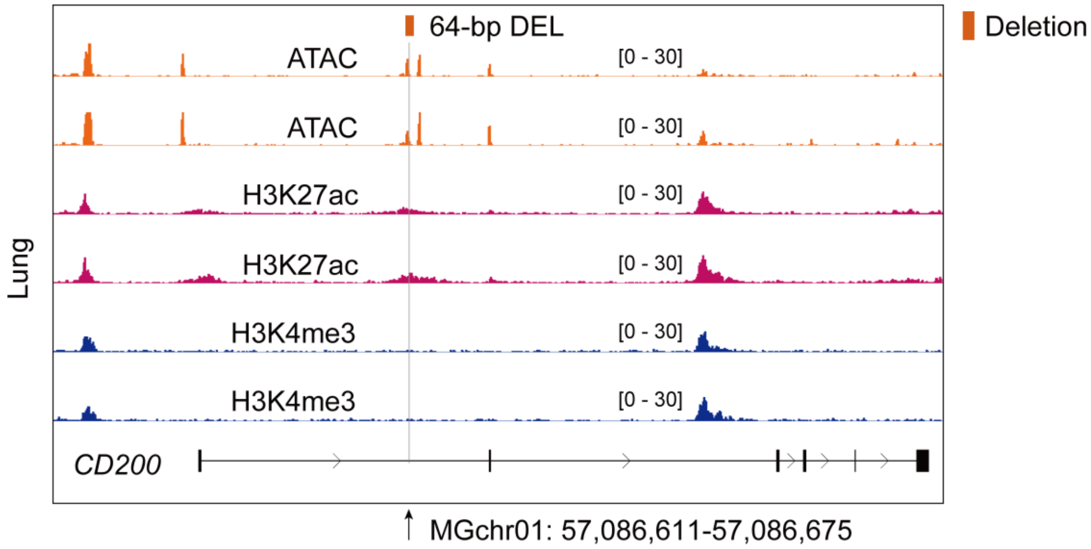

Fig. S18. An example of a 64-bp deletion (DEL) (BTA01:57,086,611-57,086,675, Mongolian_v1) located in the enhancer of *CD200* gene, which with high allele frequency difference (DI_{SV} = 0.635) and F_{ST}-SV (0.588) between northern and southern Chinese cattle. The numbers in brackets located in the tracks of ATAC-seq and ChIP-seq (H3K4me3 and H3k27ac) indicate signal intensities.

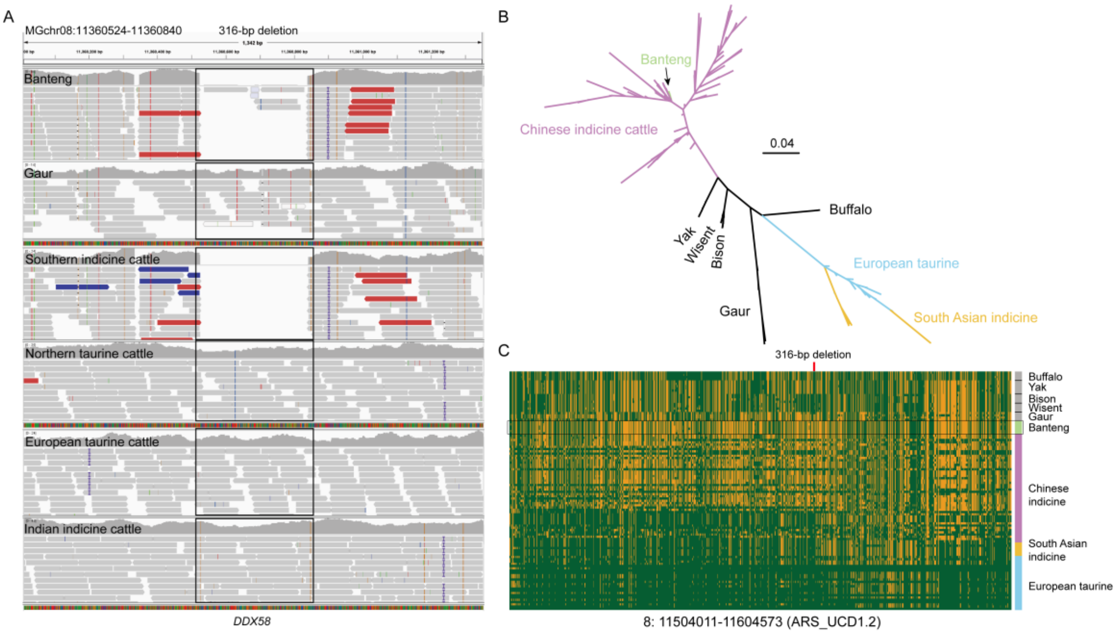

Fig. S19. A 316-bp DEL of *DDX58* might be derived from banteng. (A) IGV validation of 316-bp DEL of *DDX58* in Illumina short reads. (B) Maximum-likelihood phylogeny of segments surrounding SV on BTA08 (BTA08:11504011-11604573, ARS_UCD1.2). (C) Haplotype pattern of the segments surrounding SV on BTA08 (BTA08:11504011-11604573, ARS_UCD1.2).

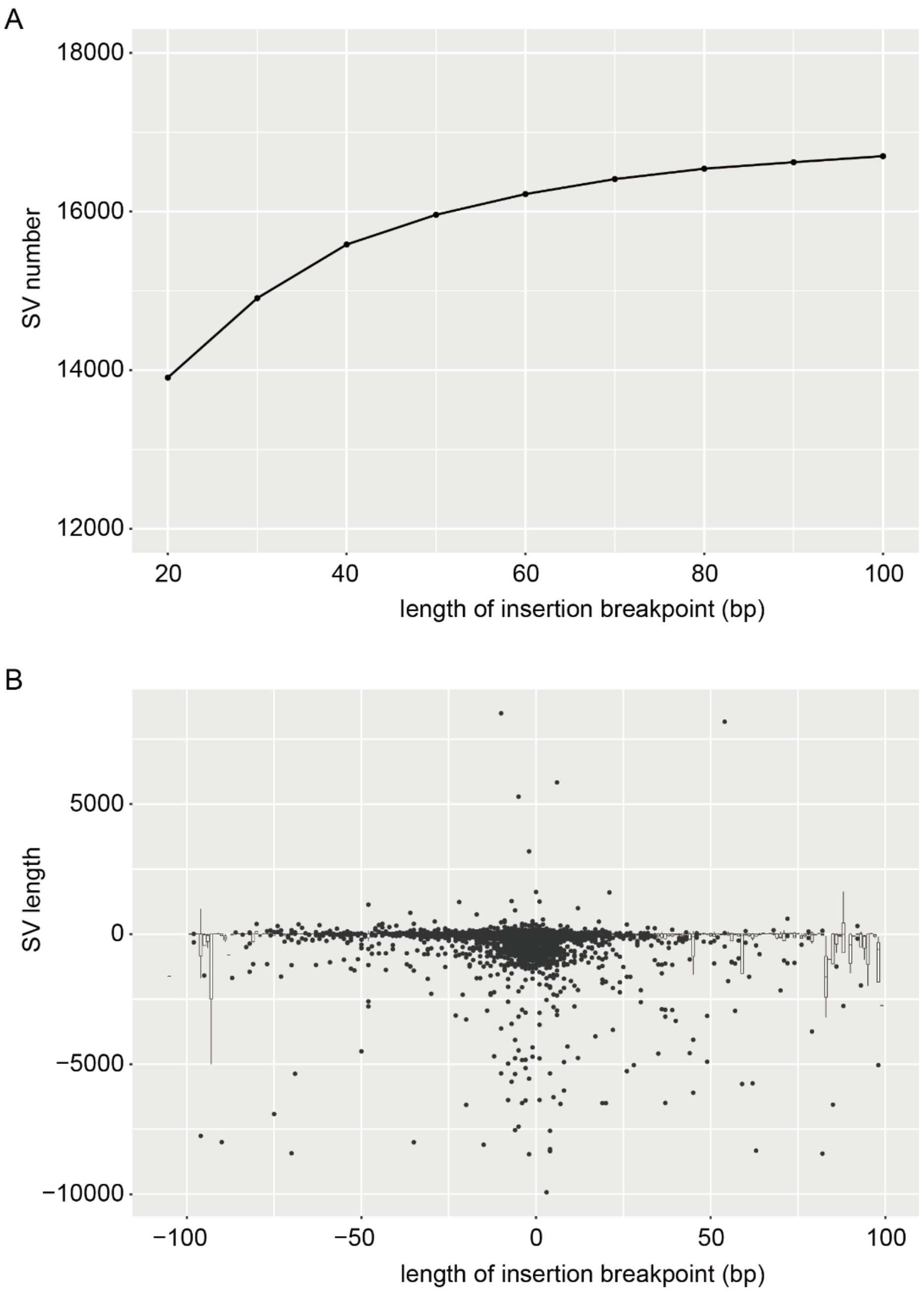

Fig. S20. Breakpoint judgement for insertions. (A) The trend of the SV number with the length of the insertion breakpoint; (B) The trend of the SV length with the length of the insertion breakpoint.
