# Supplementary material for: Structural variation and introgression from wild populations in East Asian cattle genomes confer adaptation to local environment
Source: Genome Biol. 2023 Sep 18;24:211. doi: 10.1186/s13059-023-03052-2 (PMC10507960; doi:10.1186/s13059-023-03052-2)
Supplement: Supplementary file 3 — Additional file 3. Review history. [file 13059_2023_3052_MOESM3_ESM.docx]

**Review history**

**First round of review**

**Reviewer 1**

In this study, Xia and co-authors generated two genome assemblies by integrating Nanopore long-read, Illumina short read and Hi-C data in two East Asian cattle breeds (i.e., Mongolian and Hainan), and then integrated long-read and short-read sequence data to explore the impacts of structural variants on environmental adaptation. The manuscript is in good shape. The resource will be valuable to the animal genetics community. However, after a quick search, I think the data are not publicly available yet. I appreciate if the authors could make the data publicly available at some point. The findings in general are interesting, but they might not be solid enough to support the main conclusion, i.e., the unique contributions of SVs in East Asian cattle in environmental adaptation and disease resistance. To answer this, it would be better to fully compare SV-based results with SNP-based results. For instance, what is the proportion of SVs were or were not tagged well by SNPs? The SV-genotyped population is a bit too small to infer the consequence of SV on environmental adaptation, as there are only 95 samples from nine cattle breeds, and the estimation of the allele frequency of thses SVs is challenging. Right now, just a list of SV-overlapped genes were reported here. As several SVs have been proposed to be promising candidate variants, it would be good to explore their potential functional consequence by integrating other omics data (e.g., ChIP-Seq and RNA-Seq), and associate them with some phenotypes (e.g., disease resistance) by examining Animal QTLdb. An independent validation or functional follow-ups of one promising SV could help a lot. Please see my specific comments below, hope they were helpful.

Line 58: Is the overlapping between long-read SVs and specific genes (e.g., epidermal differentiation and bovine TB) statistically significant?
Line 61: In total, how many genes do these 5563 SVs represent?
Line 62: Is this enrichment statistically significant?
Line 63: A promising example of indicine-stratified SV maybe helpful
Line 64-65: as I mentioned above the results may not be that strong to support this conclusion.
Line 91: a citation maybe helpful.
Line 119: compared to ARS-UCD 1.2, the overall size of two new assemblies is smaller. Could the authors explain this a bit? Technical or biological difference?
Line 179: Was the Fst of LRS-based SVs derived in only 9 Hainan vs. 9 Mongolian samples? The sample size seems too small to represent the populations, and the estimation of the allele frequency of SVs is challenging.
Line 235: Could the author explain this observation a bit?
Line 203: Was the PCR validation conducted in the independent samples?
Line 246: Please give the full name of GO, as it appears at the first time here.
Methods: Well described. However, it would be good that the authors could share their codes in e.g., Github for others to reproduce the findings later.

**Reviewer 2**

The manuscript is really good! The results are relevant to the field and the methods are appropriate. I'd recommend to avoid using plural for shortcuts like SVs or SNPs when you use the plural for the definition like: line 48: Structural variants (SVs). It should be SV then, please correct it throughout the manuscript for all shortcuts during the publication. Also the correct use of the subspecies name is Bos taurus taurus and Bos taurus indicus, make sure to change this whenever it appears in the manuscript. Finally, try to reduce the results section if possible! Congrats.

**Reviewer 3**

This is a very well written and straightforward paper reporting the chromosome scale assemblies of two cattle genomes (Mongolian and Hainan, taurus and indicus), and additionally long read sequencing of 18 genomes, and short read sequencing of 95 genomes and subsequent identification and characterization of structural variants. The study was conducted to specifically look at genome evolution among East Asian taurine and indicine cattle, which are relatively under studied with respect to SVs. I see no major issues with the paper. Analyses are generally appropriate and conclusions supported by data. I have only minor comments:

1) The classification and counts of SVs depend on the choice of reference genome. Please indicate throughout the manuscript that the numbers are with respect to which genome. For example, line 159. The overlap between SVs identified by whole genome alignment and by LRS based SV discovery are not comparable because the latter involves 18 genomes in total. What happens if you use the SV discovery method on the LRS data from the Hainan cattle that was used for de novo assembly and ask if the methods identify the same variants. Down-sample the reads such that they are comparable with the 18 LRS genomes.

2) Line 173, point out that the shared SVs are just singleton SV in the Mongolian reference genome.

3) The paper will benefit from comparing all SV analyses with SNP-based analyses. For example, in the population differentiation analysis (line 178), perform also SNP based analysis. In addition, for the 95 genome analysis, also do SNP based analysis to see if the same genes are identified.

4) The enrichment analysis (Figure 4c) needs to be put in the context of SV discovery. Is such enrichment specific SVs only? That's why doing a SNP based analysis is useful (see 3 above). Same goes of the banteng introgression analysis.

**Authors’ response to reviewers**

Response to Reviewers’ comments
[Comment of Reviewer #1]
In this study, Xia and co-authors generated two genome assemblies by integrating Nanopore long-read, Illumina short read and Hi-C data in two East Asian cattle breeds (i.e., Mongolian and Hainan), and then integrated long-read and short-read sequence data to explore the impacts of structural variants on environmental adaptation. The manuscript is in good shape. The resource will be valuable to the animal genetics community. However, after a quick search, I think the data are not publicly available yet. I appreciate if the authors could make the data publicly available at some point.
Response: Thank you for the comments!
We have uploaded all new sequencing data and the assembled genomes to NCBI and the data has been released. Please check the BioProject IDs of PRJNA810280, PRJNA810300, and PRJNA823479.
[Comment of Reviewer #1]
The findings in general are interesting, but they might not be solid enough to support the main conclusion, i.e., the unique contributions of SVs in East Asian cattle in environmental adaptation and disease resistance. To answer this, it would be better to fully compare SV-based results with SNP-based results. For instance, what is the proportion of SVs were or were not tagged well by SNPs?
Response: Thank you for this valuable comment!
In the revised manuscript, we have added a comprehensive comparison between SV- and SNP-based results in two aspects as follows:
(1)First, we investigated the intensity of linkage between individual SVs and surrounding SNPs (300 SNPs flanking the SVs) and realized that only 62% of the SVs had high linkage disequilibrium (LD) with surrounding SNPs (r2 > 0.8), suggesting the presence of hidden genetic variants that were only captured by SNPs (Fig. 4B). Please see lines 267-270.
(2)Second, we evaluated whether the SNPs surrounding the 2,610 stratified SVs were also differentiated based on the FST-SNP in 5-kb non-overlapping sliding windows and observed that averaged FST-SNPs values surrounding d these stratified SVs were significantly higher than those surrounding all SVs (120,006 autosomal SVs, Wilcoxon rank-sum test P < 2.22e-16). However, the FST-SNPs surrounding of the most stratified SVs (2,608, 99.92%) were below the 90th quantile (0.57) of the FST-SNPs (Fig. 4E), indicating that these SVs would probably be missed by traditional SNP-based analyses. Please see lines 291-297 and Figure 4B.
[Comment of Reviewer #1]
The SV-genotyped population is a bit too small to infer the consequence of SV on environmental adaptation, as there are only 95 samples from nine cattle breeds, and the estimation of the allele frequency of these SVs is challenging.
Response: Thank you for this valuable comment!
In the revised manuscript, we have increased the sample size of the whole-genome-sequencing data from 95 to 373 samples of 39 representative breeds/populations around the world (~15× coverage). Please see Table S13.
[Comment of Reviewer #1]
Right now, just a list of SV-overlapped genes were reported here. As several SVs have been proposed to be promising candidate variants, it would be good to explore their potential functional consequence by integrating other omics data (e.g., ChIP-Seq and RNA-Seq), and associate them with some phenotypes (e.g., disease resistance) by examining Animal QTLdb.
Response: Thank you for this valuable suggestion!
In the revised manuscript, we added experimental validation of a candidate SV within the SPN gene and used the ATAC-seq and ChIP-seq data to search for potential candidate SVs in the regulatory regions. Please see lines 300-316! We also searched Animal QTLdb (https://www.animalgenome.org/cgi-bin/QTLdb/index) as suggested. We found 41 QTLs overlapping with SVs related to milk production, behaviour, body weight/height, muscle development, etc. One of these QTLs is related to disease resistance (bovine respiratory disease susceptibility) and overlaps with a 91-bp deletion in the intron of the X-chromosomal SLC6A14 gene). However, the relevant literature on this QTL is not conclusive, so we have not discussed this in the revised manuscript.
[Comment of Reviewer #1]
An independent validation or functional follow-ups of one promising SV could help a lot. Please see my specific comments below, hope they were helpful.
Response: Thank you for this thoughtful comment!
As mentioned above, we verified the function of the 108-bp insertion in the exon of the SPN gene. We found that the 108-bp insertion in the exon of SPN has affected the uptake of Mycobacterium tuberculosis by macrophages through increased number of O-linked glycosylation sites, which may explain the low susceptibility of Hainan cattle to bovine tuberculosis.
We added the follow text: “Indicine cattle have evolved a stronger genetic resistance to ticks, parasite transmission, and other tropical diseases than taurine cattle [36]. We identified a specific 108-bp INS in the single exon of SPN (sialophorin, CD43) that encodes with an additional repeat of 36 amino acids in its extracellular domain (Fig. 3A-3B). This INS structure was validated by both IGV (Fig. S10) and PCR (Fig. S11). Other bovine species had only four to six of such repeats (Fig. S12). CD43 was found to be associated with the antigen-specific activation of T cells [37] and to inhibit the growth of Mycobacterium tuberculosis in humans [38]. CD43-deficient mice had a reduced ability to control M. tuberculosis growth during the acute and chronic phases of infection [38].
The CD43 extracellular domain has a rod-like structure that is rich in serine or threonine residues modified by heavily sialylated O-linked glycans and is extended from the macrophage cell surface, leading to mycobacterial-specific recognition through binding to the M. tuberculosis capsule protein Cpn60.2 [38, 39]. To investigate whether the increased number of O-linked glycosylation sites in CD43 affected the uptake of M. tuberculosis by macrophages, we transfected WT-SPN (wild-type, pCMV-WT-SPN) or HN-SPN (Hainan-type, pCMV-HN-SPN) into the RAW264.7 (ECACC 91062702) macrophage cell line 24 h before the infection by the H37Ra strain of M. tuberculosis at a range of multiplicity of infection (MOI) for 4 h (Fig. 3C). We found that the overexpression of CD43 resulted in significantly enhanced uptakes of M. tuberculosis by the pCMV-HN-SPN relative to the pCMV-WT-SPN (Fig. 3D) across a wide range of MOI at 5:1, 10:1, 20:1, 30:1, and 50:1 (Fig. 3E). Similar to the observation that CD43 absence reduced the production of TNF-α, IL-12, and IL-6 in the macrophages infected by M. tuberculosis [40], we detected significantly higher TNF-α and IL-6 in the RAW264.7 cells transfected by pCMV-HN-SPN compared with pCMV-HN-SPN, after the infection by H37Ra strain at an MOI of 10:1 (Fig. 3F). Because TNF-a is crucial in restricting the growth of M. tuberculosis, we speculated that the 108-bp INS in the SPN gene was related to the low susceptibility of indicine cattle to bovine tuberculosis.” Please see lines 216-240.
[Comment of Reviewer #1]
Line 58: Is the overlapping between long-read SVs and specific genes (e.g., epidermal differentiation and bovine TB) statistically significant?
Response: Thank you for the comment！
The overlapping referred to the overlap of SVs and genes in their genomic locations, therefore no statistical analysis was attempted. To avoid the ambiguity caused by this sentence, we revised this sentence to "Several SVs detected from long reads are in exons of genes associated with epidermal differentiation, skin barrier, and bovine tuberculosis resistance.". Please see lines 58-59.
[Comment of Reviewer #1]
Line 61: In total, how many genes do these 5563 SVs represent?
Response: Thank you for this specific comment!
In the revised version, we have improved the method of calculating the population stratified SV based on SRS data from sliding window to each SV locus as described by Li et al. (2023, doi: 10.1101/gr.277372.122).
Therefore, we modified the relevant content of this sentence in the Abstract according to the requirements: “Genotyping in 373 whole genome sequences from 39 breeds by short-read sequencing identified 2,610 SVs that were differentiated along a north–south gradient in China, involving 862 genes, many of which were enriched in pathways related to environmental adaptation (corrected P value < 0.05).” Please see lines 63-66.
[Comment of Reviewer #1]
Line 62: Is this enrichment statistically significant?
Response: Thank you for the comment!
A corrected P value < 0.05 was used to filter the significantly enriched pathways (please see line 283). In the revised version of the Abstract, we have added the significance description as suggested. And the content is added as follows:
“Genotyping in 373 whole genome sequences from 39 breeds by short-read sequencing identified 2,610 SVs that were differentiated along a north–south gradient in China, involving 862 genes, many of which were enriched in pathways related to environmental adaptation (corrected P value < 0.05).” Please see line 66.
[Comment of Reviewer #1]
Line 63: A promising example of indicine-stratified SV maybe helpful
Response: Thank you for this valuable comment!
In the revised Abstract, we present an example of a 316-bp deletion in the DDX58 gene, which has a high frequency in Chinese indicine cattle (53.3%). The content is added as follows:
“We identified 1,457 Chinese indicine-stratified SVs that possibly originated from banteng, among which a 316-bp intronic deletion in DDX58 was frequent in Chinese indicine cattle.” Please see lines 66-68.
[Comment of Reviewer #1]
Line 64-65: as I mentioned above the results may not be that strong to support this conclusion.
Response: Thank you for this thoughtful comment!
To support the conclusion of “the unique contributions of SVs in East Asian cattle to environmental adaptation and disease resistance”, we selected the SV in the SPN gene as a candidate SV for verification. SPN was found to be associated with the antigen-specific activation of T cells and to inhibit the growth of Mycobacterium tuberculosis in humans. We demonstrated that a 108-bp insertion in the exon of SPN may affect the uptake of M. tuberculosis by macrophages through increased number of O-linked glycosylation sites, which plausibly is related to the low susceptibility of Hainan cattle to bovine tuberculosis. Please see lines 216-240.
[Comment of Reviewer #1]
Line 91: a citation maybe helpful.
Response: Thank you for your comment!
We have added two relevant citations as suggested. Please see line 96.
[Comment of Reviewer #1]
Line 119: compared to ARS-UCD 1.2, the overall size of two new assemblies is smaller. Could the authors explain this a bit? Technical or biological difference?
Response: Thank you for this comment
This may be mainly a technical difference. We evaluated the assembly completeness of the three genomes (Mongolian_v1, Hainan_v1, and ARS_UCD1.2) by different methods, and finally concluded that the completeness of the ARS_UCD1.2 reference genome was higher. For example, the comparison rate between BUSCO evaluation (please see Table S2) and the number of SNPs in the population-specific genome (please see lines 156-158) suggest that the higher completeness of ARS_UCD1.2 has more impact on variant detection than the allelic bias of the genome.
[Comment of Reviewer #1]
Line 179: Was the Fst of LRS-based SVs derived in only 9 Hainan vs. 9 Mongolian samples? The sample size seems too small to represent the populations, and the estimation of the allele frequency of SVs is challenging.
Response: Thank you for this valuable comment!
The FST in Line 179 is the result of 18 LRS-based SVs (9 Hainan vs 9 Mongolian). Considering that the SV set identified in the LRS data is more accurate, this study uses Hainan and Mongolian cattle as the representative breeds to call SV, and then includes the SRS data of 373 cattle to expand the sample size for verification. Calling SVs with LRS data has a significant advantage, especially for some important SVs that cannot be verified in the short Illumina sequencing data, for instance, the 108-bp insertion in the SPN gene. With a further development of sequencing technology and a decrease in sequencing cost, expanding the LRS data from larger sample sizes to detect SV will allow us to generate a more complete SV data set.
[Comment of Reviewer #1]
Line 235: Could the author explain this observation a bit?
Response: Thank you for this suggestion!
In the revision, we repeated the PCA analysis for different reference genomes of 39 cattle breeds/populations and found that the SNP calls based on the Hainan_v1, Mongolian_v1, and ARS_UCD1.2 assemblies yielded the same PCA patterns (please see Fig. S15). Therefore, our previous conclusion "Calling SNPs based on the Hainan_v1 and ARS_UCD1.2 assemblies yielded different PCA patterns, but these still differentiated northern and southern Chinese cattle", which was based on 95 individuals, has been omitted in the revised manuscript.
[Comment of Reviewer #1]
Line 203: Was the PCR validation conducted in the independent samples?
Response: Thank you for this valuable comment.
The SV in the SPN gene has now been PCR-verified in additional 40 Hainan and 37 Weizhou (a Chinese indicine breed) cattle samples. For Fig. S11, we selected five individuals carrying the three SV genotypes.
[Comment of Reviewer #1]
Line 246: Please give the full name of GO, as it appears at the first time here.
Response: Thank you for this thoughtful comment!
In the revised manuscript, we have added the full name of GO. Please see line 283.
[Comment of Reviewer #1]
Methods: Well described. However, it would be good that the authors could share their codes in e.g., Github for others to reproduce the findings later.
Response: Thank you for this suggestion!
As requested, we uploaded the scripts and pipelines we used at GitHub (https://github.com/K-zhangfengwei/Structural_variation_in_East_Asian_cattle). Please see lines 655-656.
[Comment of Reviewer #2]
The manuscript is really good! The results are relevant to the field and the methods are appropriate. I'd recommend to avoid using plural for shortcuts like SVs or SNPs when you use the plural for the definition like: line 48: Structural variants (SVs). It should be SV then, please correct it throughout the manuscript for all shortcuts during the publication. Also the correct use of the subspecies name is Bos taurus taurus and Bos taurus indicus, make sure to change this whenever it appears in the manuscript. Finally, try to reduce the results section if possible! Congrats.
Response: Thank you for your comments!
(1) As requested, we have corrected all the shortcuts used for definitions. Please see line 48: Structural variations (SV); line 94: single nucleotide polymorphisms (SNP), insertions (INS), deletions (DEL), inversions (INV), and duplications (DUP); line 140: long interspersed nuclear element (LINE) and short interspersed nuclear element (SINE).
(2) In the revised version, we have corrected the subspecies names as suggested. Please see lines 75-76 and 195-196.
In this revised manuscript, we have reduced the most speculative parts from the Results section but presented addition experimental verification in the revised version.
[Comment of Reviewer #3]
This is a very well written and straightforward paper reporting the chromosome scale assemblies of two cattle genomes (Mongolian and Hainan, taurus and indicus), and additionally long read sequencing of 18 genomes, and short read sequencing of 95 genomes and subsequent identification and characterization of structural variants. The study was conducted to specifically look at genome evolution among East Asian taurine and indicine cattle, which are relatively under studied with respect to SVs. I see no major issues with the paper. Analyses are generally appropriate and conclusions supported by data. I have only minor comments:
1) The classification and counts of SVs depend on the choice of reference genome. Please indicate throughout the manuscript that the numbers are with respect to which genome. For example, line 159.
Response: Thank you for the comment!
We apologize for the missing information on the reference genome. In fact, both the whole-genome alignment and LRS-SV are based on the Mongolian_v1 assembly. We have added a description of the reference genome used in each of the corresponding places in the revised manuscript. Please see lines 164 and 338.
[Comment of Reviewer #3]
The overlap between SVs identified by whole genome alignment and by LRS based SV discovery are not comparable because the latter involves 18 genomes in total. What happens if you use the SV discovery method on the LRS data from the Hainan cattle that was used for de novo assembly and ask if the methods identify the same variants. Down-sample the reads such that they are comparable with the 18 LRS genomes.
Response: Thank you for this valuable comment!
According to the comparison scheme proposed by the reviewer, we have revised the manuscript content. We compared the numbers of SVs obtained based on the whole-genome alignment and LRS data (11.02 ×) from the same samples of HN024 for Hainan_v1 and NMG016 for Mongolian_v1, and identified 38,254 (19,765 INSs and 18,489 DELs) and 23,554 (7,974 INSs and 15,580 DELs) INDELs > 50 bp, respectively. A total of 17,756 SVs were overlapped between the two methods, including 6,293 INSs and 11,463 DELs. The higher INDELs from the whole-genome alignment were attributed to the higher accuracy of assembly-based SVs [25], which is consistent with previous results [26]. More DELs were observed in the LRS data due possibly to base-calling errors of homopolymer regions, a major drawback of Nanopore sequencing [27]. Please see lines 176-184.

[Comment of Reviewer #3]
2) Line 173, point out that the shared SVs are just singleton SV in the Mongolian reference genome.
Response: Thank you for this suggestion!
As observed previously by Audano et al. (2019; Characterizing the Major Structural Variant Alleles of the Human Genome. Cell. 176663-675.), we mention that the shared and major SVs may be the result of minor alleles or assembly errors in the Mongolian_v1 genome. Therefore, we added to the revised version: “Approximately 31.6% (n = 34,164) of the LRS-based SVs were shared or major, which indicating that the Mongolian_v1 reference genome carries a minor allele or an error at these locations as observed previously for human assemblies [28]. Among the shared SVs, DELs were more abundant than INSs (Fig. 2B and 2C), which was also consistent with the results obtained in humans [29].”
Please see lines 187-191.
[Comment of Reviewer #3]
3) The paper will benefit from comparing all SV analyses with SNP-based analyses. For example, in the population differentiation analysis (line 178), perform also SNP based analysis. In addition, for the 95 genome analysis, also do SNP based analysis to see if the same genes are identified.
Response: Thank you for this valuable comment！
In addition, we performed an FST analysis using the genome-wide SNPs from the same populations to see how many genes identified from top FST-SNPs values (e.g., top 5%) were overlapped with FST-SV genes. Up to 61%, 53%, 44%, 32%, and 25% of the genes were found in the non-overlapping sliding windows of 5 kb, 10 kb, 20 kb, 50 kb and 100 kb, respectively. Please see lines 287-290.
In addition, we added a comprehensive comparison between SV-based results and SNP-based results, which also was requested by Reviewer 1:
(1)First, we investigated the intensity of linkage between individual SVs and surrounding SNPs (300 SNPs flanking the SVs) and realized that only 62% of the SVs had high linkage disequilibrium (LD) with surrounding SNPs (r2 > 0.8), suggesting the presence of hidden genetic variants that were only captured by SNPs (Fig. 4B). Please see lines 267-270.
(2)Second, we evaluated whether the SNPs surrounding the 2,610 stratified SVs were also differentiated based on the FST-SNP in 5-kb non-overlapping sliding windows and observed that averaged FST-SNPs values surrounding d these stratified SVs were significantly higher than those surrounding all SVs (120,006 autosomal SVs, Wilcoxon rank-sum test P < 2.22e-16). However, the FST-SNPs surrounding of the most stratified SVs (2,608, 99.92%) were below the 90th quantile (0.57) of the FST-SNPs (Fig. 4E), indicating that these SVs would probably be missed by traditional SNP-based analyses. Please see lines 291-297 and Figure 4B.
[Comment of Reviewer #3]
4) The enrichment analysis (Figure 4c) needs to be put in the context of SV discovery. Is such enrichment specific SVs only? That's why doing a SNP based analysis is useful (see 3 above). Same goes of the banteng introgression analysis.
Response: Thank you for this valuable comment!
In the revised version, we have improved the method of calculating the population-stratified SV. Specifically, DISV and FST distances were used to search for SVs differentiating between southern and northern cattle, and 2,610 ‘north–south’ SVs were obtained. Among these, 1,070 SVs potentially affect 862 genes based on their presence in exons (10), UTRs (18), upstream (<1 kb, 14), intergenic (<3 kb, 27) and introns (1,001). These 862 genes were found to be enriched in genes involved signaling pathways that might be associated with ‘north–south’ SV. Please see lines 279-286. In addition, we replaced an extensive descriptions of pathway enrichment by ATAC-seq and ChIP-seq data to further identify candidate SVs that in regulatory regions.
Taking advantage of the RFMix analysis results, we found that 99.4% (1,457/1,466) of the introgressed SVs are within introgressed regions that were identified on the basis of SNPs, indicating a high consistency between the results of SV and SNP. Please see lines 330-332. Taking 316-bp DEL in the DDX58 gene as an example, we constructed a phylogenetic tree in 46 individuals to be homozygous for the introgressed SVs, to infer the origin of haplotypes based on the SNPs surrounding the SVs (Fig. S19). The results showed that the 316-bp DEL was indeed located within the region of banteng-introgressed SNPs. However the origin of southern Chinese cattle is rather complex, the impact of other bovine species on Chinese indicine cattle cannot be completely excluded, including the extinct (kouprey) or other unsampled wild bovine species or lineages. Please check lines 405-413 for a discussion of the results in this section.

**Second round of review**

**Reviewer 1**

I had a good look at the response letter and resvised manuscript. Most of my comments have been addressed and I do not have any further comments.

**Reviewer 3**

The authors have adequately addressed all my concerns.
